# Mulberry Leaf Regulates Differentially Expressed Genes in Diabetic Mice Liver Based on RNA-Seq Analysis

**DOI:** 10.3389/fphys.2018.01051

**Published:** 2018-08-07

**Authors:** Qi Ge, Shu Zhang, Liang Chen, Min Tang, Lanlan Liu, Mengna Kang, Lu Gao, Shangshang Ma, Yanhua Yang, Peng Lv, Ming Kong, Qin Yao, Fan Feng, Keping Chen

**Affiliations:** ^1^Institute of Life Sciences, Jiangsu University, Zhenjiang, China; ^2^School of Food and Biological Engineering, Jiangsu University, Zhenjiang, China; ^3^Department of Genetics and Genomic Sciences, Icahn School of Medicine at Mount Sinai, New York City, NY, United States; ^4^The Fourth Affiliated Hospital of Jiangsu University, Zhenjiang, China

**Keywords:** mulberry leaf powder, diabetes mellitus, gene expression, RNA-Seq, transcript level, qRT-PCR, Western blotting

## Abstract

The pathogenesis of diabetes mellitus is a complicated process involving much gene regulation. The molecular mechanism of mulberry (*Morus alba* L.) leaf in the treatment of diabetes is not fully understood. In this study, we used the Illumina HiSeq™ 2,500 platform to explore the liver transcriptome of normal mice, STZ-induced diabetic mice, and mulberry leaf-treated diabetic mice, and we obtained 52,542,956, 52,626,414, and 52,780,196 clean reads, respectively. We identified differentially expressed genes (DEGs) during the pathogenesis of diabetes in mice. The functional properties of DEGs were characterized by comparison with the GO and KEGG databases, and the results show that DEGs are mainly involved in the metabolic pathway. qRT-PCR was used to analyse 27 differential genes involved in liver expression in different groups of diabetic mice. Among the DEGs, the expression of *Scube1, Spns3, Ly6a, Igf2*, and other genes between the control (C) and diabetic control (DC) groups was significantly upregulated; the expression of *Grb10, Mup2*, and *Fasn* was significantly downregulated; the expression of the *Sqle, Lss*, and *Irs2* genes between the C group and diabetic group treated with mulberry (DD) was significantly upregulated; the expression of *Fabp2, Ly6a*, and *Grb10* was significantly downregulated; and the expression of *Sqle* and *Lss* was significantly upregulated in the DC and DD groups, but *Tap1, Igf2*, and *Spns3* were significantly downregulated. The results of Western blot validation showed that dynamic changes in proteins, such as IGF2, Ly6a, Grb10, and UBD, occurred to regulate the incidence of diabetes by influencing the insulin receptor substrate (IRS) signaling pathway.

## Introduction

Diabetes mellitus (DM) is a chronic metabolic disease characterized by elevated blood glucose levels and metabolic disorders of glucose, fat, and protein in the body (Ge et al., 2017). Diabetes is caused by a lack of insulin secretion or insulin resistance and low utilization of sugar, which then can lead to brain, heart, kidney, heart failure, acute myocardial infarction (Ong et al., 2018) and other organ complications that can severely harm human health (Davis et al., 2006; Nie et al., 2017). The relevant data indicate that the current global total number of diabetic patients is close to 300 million, and the number of diabetic patients is still increasing (Kwak and Park, 2016). Obesity is considered as a major factor to develop type 2 diabetes mellitus (T2DM), and controlling weight gain is very important to prevent and treat diabetic disorders (Yan et al., 2016). High insulin levels in patients with an abnormal glucose regulation mechanism caused by glycosuria phenomenon must be treated to prevent the development of corresponding complications (Michels and Eisenbarth, 2011).

The precise etiology of diabetes is very complex and includes genetic factors that may lead to diabetes. The genetic characteristics are the risk characteristics for diabetes and in the context of external environment and institutional characteristics, diabetes will be induced (Chang et al., 2013). Parkkola et al. found that human leukocyte antigen (*HLA*) genes are involved in the pathogenesis of type I diabetes (Parkkola et al., 2017). HLA is a major histocompatibility complex (MHC) that presents antigens to T cells, which can identify the antigen, that are secreted by the B cells. Under normal circumstances, HLA is expressed only in the surface of B lymphocytes, activated T lymphocytes, macrophages, and endothelial cells, and the surface expression of islet beta cells is associated with autoimmune disease (Odermarsky et al., 2017). Type II diabetes mellitus, which is different from type I diabetes mellitus, involves basal insulin secretion and basal insulin sensitivity abnormalities caused by the inheritance of multiple recessive genes. Gao et al. and Huang et al. examined the *ins* gene in type II diabetic patients and found that the expression of the *ins* gene in type II diabetes patients was changed compared with that in normal patients and other illnesses, indicating that the change of the *ins* gene may be one of the causes of type II diabetes (Gao et al., 2016; Huang et al., 2017). In addition to genetic factors, diabetes mellitus is associated with insulin resistance. Insulin resistance refers to the role of insulin in promoting glucose uptake resistance, with an increase in secondary compensatory insulin secretion, which can produce a series of adverse effects and a variety of pathophysiological changes to the body, and it has become the common basis for the development of some diseases (Mlinar et al., 2007).

Thus far, although many drugs for the treatment of diabetes have been developed, almost all of them are chemical or biological agents, such as sulfonylureas (Carvalho-Martini et al., 2006) and biguanides (Okamoto et al., 2007). Biguanides are used to promote glucose uptake by peripheral tissues of muscle, inhibit glucose intolerance, and delay glucose absorption in the gastrointestinal tract, and the combined use of sulfonylurea with biguanides may enhance the hypoglycemic effect. However, due to additional complications of diabetes, and because it cannot be permanently cured, only drug-based treatment can be used to control the disease through the hypoglycemic effect. The drugs for the treatment of type II diabetic patients exert a certain curative effect, but they also have some negative side effects, and long-term administration can be detrimental to the physiology and psychology of the patients.

In traditional Chinese medicine (TCM), certain herbs have been used for diabetes. Sarikaphuti et al. demonstrated that anthocyanins extracted from *Morus alba* L. were well tolerated and exhibited effective anti-diabetic properties in diabetic rats (Sarikaphuti et al., 2013). Ou et al. suggest that the mulberry extract may be active in the prevention of fatty liver (Ou et al., 2011). Wu et al. determined that cherry anthocyanin and mulberry anthocyanin can alleviate oxidative stress and inflammation associated with developing obesity in mice fed with high-fat diet (Wu et al., 2016). Therefore, natural herbs are receiving increasing attention, including mulberry leaves (Jouad et al., 2001). Mulberry leaf possesses many pharmacological effects, which has been traditionally used in Chinese medicines for diuretics, liver protection, eyesight improvement, blood pressure reduction, and cardiovascular disease prevention.

Mulberry is widely planted in China, and includes white mulberry (*Morus alba*), red mulberry (*Morus rubra*), black mulberry (*Morus nigra*) etc.; their leaves and extracts are used as a natural source of disease treatment. Previous studies (Hu et al., 2017) have noted that the white mulberry (*Morus alba* L.) leaf as a silkworm diet contains a variety of nutrients, of which flavonoids are known to possess antioxidant activity and scavenge free radicals (Proença et al., 2017). Mulberry polysaccharides had significant hypoglycemic effect with signifcantly lowering of blood glucose levels in diabetic mice (Liao et al., 2018). Many researchers at home and abroad have treated diabetes with mulberry leaves, which contain natural inhibitors of glycosidase activity. Ou et al. and Peng et al. indicated the hypolipidemic effects of mulberry water extracts on oleic acid-induced HepG2 cells and hamsters supplied with high-fat diet (Ou et al., 2011; Peng et al., 2011). Ann et al.'s studies confirmed that the main hypoglycemic active compound in mulberry leaves is alkaloid and it has the effect of reducing blood glucose in animal experiments (Ann et al., 2015). Besides, polysaccharide from mulberry plants showed a protective function on damaged pancreatic islets and β-cell (Li et al., 2011). Therefore, herbal medicines have been considered as a potential treatment for diabetes based on its safety, cost, and effectiveness (Song et al., 2009).

In this study, we used RNA-Seq transcriptomic techniques to identify differentially expressed genes in diabetic mice and mulberry leaf-treated diabetic mice and to understand the diverse functions of these differentially expressed genes. Based on our sequencing and validation results, we focused on the regulatory mechanisms of mulberry leaf powder in the treatment of diabetes through the changes in genes related to glucose metabolism in diabetic mice.

## Materials and methods

### Study approval

The study was approved by the Ethics Committee and the Scientific Investigation Board of Jiangsu University (Zhenjiang, Jiangsu Province, China). All experimental procedures were performed in accordance with the recommendations found in the Guide for the Care and Use of Laboratory Animals published by the US National Institutes of Health (NIH publication no. 85-23 revised 1996).

### Animal model construction

The specific pathogen-free (SPF)-grade Institute for Cancer Research (ICR) male mice used in this experiment were provided by the Animal Experiment Center of Jiangsu University in Zhenjiang City, Jiangsu Province, China, and housed in a fully enclosed clean animal room with alternating dark and light for 12 h, relative humidity at 40–65%, and temperature at 25 ± 1°C. SPF mouse food pellets and litter were provided by the Animal Experiment Center of Jiangsu University processing; the mice were provided with access to food and water *ad libitum*.

After 7 days of adaptive feeding, 50 male ICR mice weighing 25–30 g were randomly divided into the normal group (*n* = 10) and the model group (*n* = 40). After fasting for 12 h, the diabetic model mice were intraperitoneally injected with 150 mg/kg streptozotocin (STZ) solution; the normal mice were injected intraperitoneally with the same dose of citrate buffer. Food and water consumption and survival were observed, and the tail blood of mice was analyzed with a glucose meter to determine the fasting blood glucose after 72 h. The diabetic mouse model was successfully constructed when the fasting blood glucose measured more than 11.1 mol/L.

### Mulberry leaf powder gavage test for diabetic mice

Ten normal mice (control, group C) were selected, and 20 diabetic mice (20 mice) were randomly divided into the diabetic mouse group (diabetic control, DC group, 10 mice) and the mulberry leaf powder gavage group (DD group, 10 mice). Mulberry leaves (*Morus alba* var*. multicaulis* (Perrott.) Loud.) were harvested from a mulberry garden of Jiangsu University in Zhenjiang City, Jiangsu Province, China. The leaves from the apex of healthy plants were plucked, thoroughly washed, dried, and ground to a fine powder in an electric grinder. For treatment, 40 g of mulberry powder was dissolved in 1 L of sterilized double-distilled water to obtain a 40 mg/ml mulberry leaf powder solution. The DD mice were given mulberry leaf powder solution by intragastric administration (80 mg/kg daily) for 10 weeks, while the C group and DC group were given the same dose of double-distilled water every day for 10 weeks. Throughout the experiment, all mice were not exposed to any hypoglycemic agents (Hu et al., 2017). After 10 weeks of continuous gavage, the body weight and blood glucose of the mice in each group were measured. The mouse serum insulin content was measured according to the mouse insulin ELISA kit (Thermo Fisher Scientific, USA) instructions. The glucose tolerance test (GTT) was performed on the 10th week before the mice were killed. Briefly, the mice were intraperitoneally injected with glucose (2.0 g/kg BW, sigma) and then examined using a blood glucose meter at 0, 0.5, 1.0, 1.5, 2.0 h post injection. And the blood glucose concentration was determined after overnight fasting (12 h).

### Sample preparation and RNA extraction

None of the three groups of mice had died during the whole experiments. Six mice from each group with better growth conditions were selected, and after killing mice by cervical dislocation, the livers from the three groups of mice were rapidly removed and quickly ground in a mortar containing liquid nitrogen. All of the animal experiments were conducted in accordance with the guidelines and approval of the Animal Research and Ethics Committee of Jiangsu University.

Total RNA was extracted from approximately 0.1 g of liver powder using a TRIzol® reagent kit (Thermo Fisher Scientific, USA), according to the manufacturer's instructions. The concentration of total RNA was estimated by measuring the absorbance at 260 nm using OD_1000_, and the integrity of the 28S and 18S ribosomal RNA was detected on a 1% agarose gel. The same volume of RNA from each tissue was pooled and used for subsequent cDNA synthesis.

### cDNA library construction and illumina sequencing

Briefly, eukaryotic mRNA was enriched with magnetic beads containing Oligo (dT), and then a fragmentation buffer was used to break the mRNA into short pieces. The disrupted mRNA was used as a template to synthesize a cDNA strand with a six-base random primer, and then a two-strand synthesis reaction was prepared to synthesize double-stranded cDNA. In the cDNA second-strand synthesis, dTTP was replaced with dUTP, and then different linkers were ligated and one strand containing dUTP was digested with the UNG enzymatic method to retain only one cDNA strand of different linkers. The PureLink PCR Purification Kit (Invitrogen, Carlsbad, CA, USA) was then used to purify a single cDNA strand. The cDNA was ligated into a strand and then ligated to a sequencing adapter. A fragment size was selected, and PCR amplification was performed to construct a sequencing library. The constructed library was qualified with the Agilent 2100 Bioanalyzer, and the Illumina HiSeq™ 2500 platform was used for sequencing.

### Illumina sequence assembly and QC analysis

The raw data obtained by the Illumina sequencing platform were analyzed to determine if the sequencing data were suitable for subsequent analysis. After this quality control step, the clean reads were aligned to the reference sequence using TopHat/Bowtie2. After this comparison, the sequencing data were further refined to remove the low-quality sequences and the linker sequence by statistical distribution of the reads in the reference sequence and the coverage, thereby reducing the influence on the subsequent analysis. The data filtering criteria were: (1) filter low-quality reads with a quality threshold of 20 and a filter length threshold setting of 70%; (2) remove low-quality bases from the 3′ end with a mass threshold of 20; and (3) length threshold of 35 bp. The raw data were then mass-filtered and quality-filtered by FastQ (http://www.bioinformatics.babraham.ac.uk/projects/fastqc/) to determine whether the comparison result was properly aligned for gene expression analysis.

### Sequence assembly, and functional gene annotation and classification

For all of the original reads, low-quality base and adapter sequences were deleted, and then all of the clean reads were mapped to the reference genome via TopHat2 (version 2.0, http://www.carpbase.org/download_home.php). The gene expression was calculated using a method per kilobase exon fragment per Fragments Per Kilobase of transcript per Million fragments mapped (FPKM). The DEGs in the library were identified using the edgeR package (http://www.r-project.org/). We identified genes with fold change ≥2 (log2FC > 1) and false positive rate (FDR) < 0.05 by comparing significant DEGs using this equation:

$$\begin{array}{l} P=1-\overset{m-1}{\underset{i=0}{\sum }}\frac{\left(\begin{array}{c} M\\ i \end{array}\right)\left(\begin{array}{c} N-M\\ n-i \end{array}\right)}{\left(\begin{array}{c} N\\ n \end{array}\right)} \end{array}$$

The enrichment score was calculated as:

$$\begin{array}{l} Enrichment\;score{=}^{\frac{m}{n}}{/}_{\frac{M}{N}}, \end{array}$$

where *N* is the number of genes with GO annotation in all of the genes, *n* is the number of genes with GO annotation in differentially expressed genes in *N, M* is the number of genes annotated as a particular GO term in all of the genes, and m is the annotation of a specific GO term.

### GO and KEGG pathway analysis of DGEs

GO enrichment analysis of the differentially expressed genes was performed, and their function was described (in combination with the GO annotation results). The number of differentially expressed genes included in each GO entry was counted, and the supergeometric distribution test method was used to calculate the significance of the diversity gene enrichment in each GO entry. The calculated result returned a rich *p*-value; a small *p*-value indicates that the differentiated gene is enriched in the GO entry. Based on the results of the GO analysis, the gene of the subsequent study can be selected according to the biological significance. Pathway analysis was performed using the Kyoto Encyclopedia of Genes and Genomes (KEGG) database annotation results of differential genes, and the significance of differential gene enrichment in each pathway entry was calculated using the hypergeometric distribution test. Pathways involved in differentially expressed genes were drawn as bubble charts using the OmicShare (http://www.omicshare.com/tools/Home/Soft/senior) online tool to analyze their pathway.

### qRT-PCR to verify differential gene expression

To validate the Illumina sequencing data, 27 differential genes in the normal group, and the STZ-induced diabetic and mulberry powder-treated mice were selected for qRT-PCR analysis, and the same RNA samples were used for transcriptomic analysis. A 25-μl PCR reaction mixture was prepared using a SYBR Green PCR Kit (Qiagen, Hilden, Germany) with 20 ng of cDNA as a template. After mixing, the PCR reaction was performed using an ABI 7300 instrument. The β-actin gene was used as a housekeeping gene to normalize the expression level of the test gene, and the relative gene expression level was analyzed using the 2^−ΔΔCT^ method. All of the primers were synthesized by Shanghai Yingjun Biological Company. All primer sequences are listed in Table S5. All of the samples were analyzed in triplicate.

### Antibody preparation and western blot analysis

Tissues from each group of mice were lysed in radioimmunoprecipitation assay (RIPA) lysis buffer for 30 min. Proteins were separated by centrifugation (4°C, 12,000 × *g*, 15 min) and assayed by Bio-Rad dye binding assay. Next, 40–50 μg of protein was separated on a 10% sodium dodecyl sulfate-polyacrylamide gel electrophoresis (SDS-PAGE) gel and electrophoresed at 100 V for 90 min. The protein was then transferred to a polyvinylidene difluoride (PVDF) membrane with an electrotransfer device at 250 V for 2 h. After the transfer was completed, the PVDF membrane was blocked for 2 h in blocking solution (5% skim milk powder in Tris-buffered saline and Tween 20 (TBST), pH 7.4) to reduce non-specific binding. Then, the membrane was incubated with the primary antibody and placed on a shaker at 4°C for overnight incubation. The antibodies used were anti-ubiquitin D antibody (ab134077, Abcam, Cambridge, MA, USA), anti-GRB10 antibody (ab154029, Abcam), anti-IGF2 antibody (ab170304, Abcam), and anti-CYP51A1 antibody (ab210792, Abcam). The incubated PVDF membrane was placed in a TBST solution and washed three times for 5 min each time. The membrane was incubated for 2 h at room temperature using a fluorescent secondary antibody (1:5,000 dilution), followed by washing with TBST three times, for 5 min each time. Imaging and analysis of the data were performed using the ODYSSEY Family of Imaging Systems. The difference in the expression of genes at the protein level was detected.

### Statistical analysis

All of the experimental data are expressed as the mean ± standard error (x ± s). One-way analysis of variance (ANOVA) was used to evaluate the homogeneity variance for each group. Statistical Package for the Social Sciences (SPSS) software was used to perform the multiple comparisons between the tested groups. Statistically significant differences were defined as *P* < 0.05.

## Results

### Diabetic mouse modeling and physiological differences

**Figure 1:**
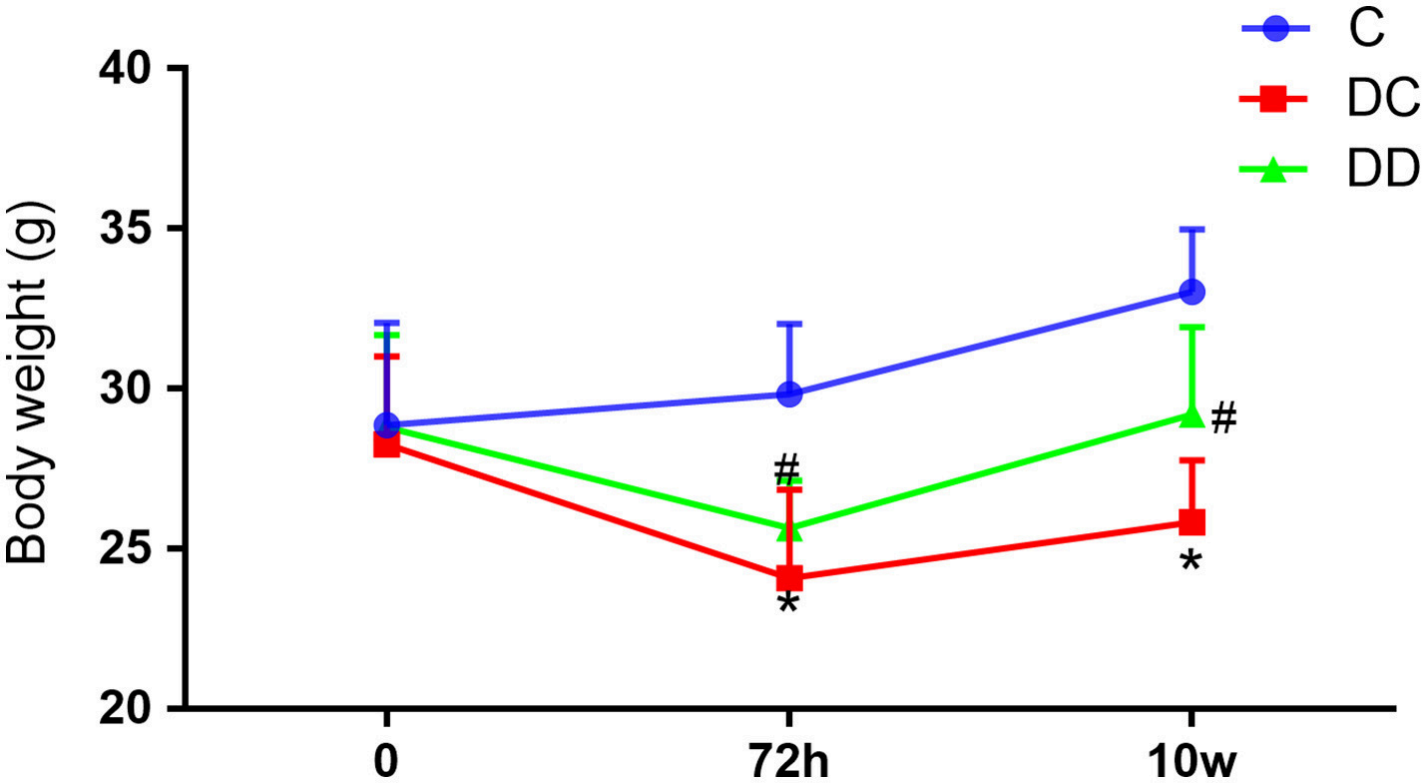
The body weight of mice in the different treatment groups at different time points. Weight loss of the diabetic mice during the trial was relatively reduced. For the diabetic mice that received mulberry leaf powder, their body weight increased. After 10 weeks, the body weight of the mulberry leaf-treated mice was significantly higher than that of the diabetic mice; the body weight of the mice that received the mulberry leaf powder was lower than that of normal mice. *P < 0.05, the incidence of the disease group compared with the normal group; ^##^*P* < 0.01, mulberry leaf powder treatment group compared with the incidence of disease group. Value = mean ± SD (*N* = 6).

Mice in the normal control group had smooth and shiny fur, a good mental state, and were more lively and responsive. The STZ-induced diabetic mice had less activity, and were apathetic and unresponsive. After the mulberry leaf powder solution treatment, the diabetic mice returned to normal. The results of body weight measurement showed that the body weight of mice in the diabetic model group was decreased after the injection of STZ solution for 72 h, and the body weight of mice in the gavage group slowly increased after the continuous use of mulberry leaf powder for 10 weeks (Figure 1; Table S1). The results speculated that the hypoglycemic mechanism of mulberry leaves involving the increase of insulin release, which is generally believed to play a role in the small intestine after oral administration and delay the absorption of carbohydrates in food, thus achieving the effect of losing weight.

**Figure 2:**
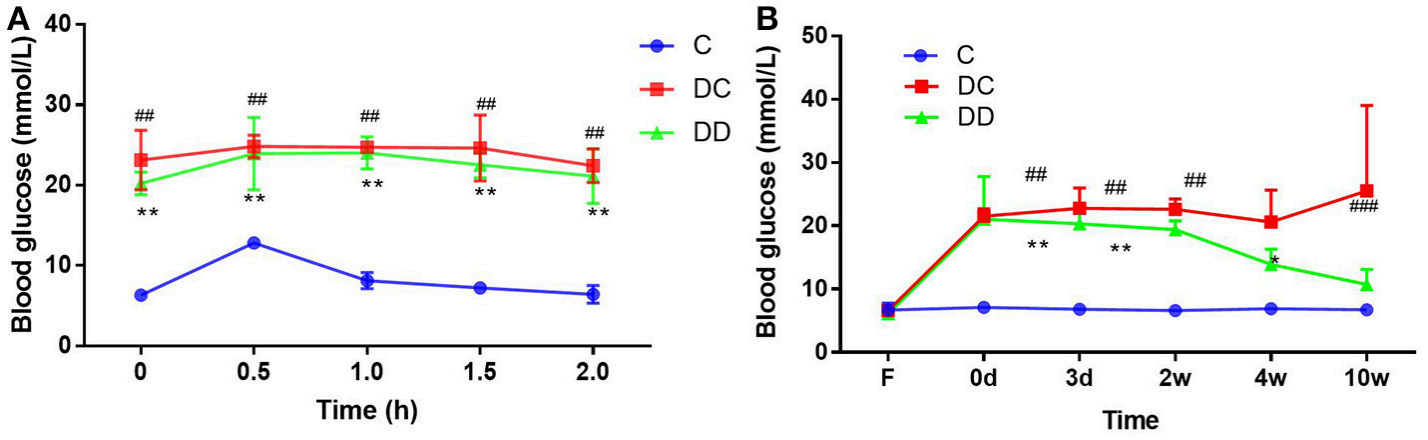
Fasting blood glucose in the three groups of mice and anti-hyperglycemic effect of mulberry leaf. **(A)** Glucose tolerance test (GTT, mmol/L), mulberry leaf enhance insulin sensitivity of diabetic mice, **P < 0.01, DD compared with C; ^##^*P* < 0.01, DC compared with C. **(B)** FBG levels of mice, FBG of DC group maintained high level throughout the trial, however, mulberry leaf could significantly reduce FBG of diabetic mice, *P < 0.05; **P < 0.01, DD compared with C; ^##^*P* < 0.01, DC compared with C.

STZ treatment successfully induced hyperglycemia in mice (Figure 2). The results of the fasting blood glucose measurement showed that the fasting blood glucose level in the diabetic model group was higher than 11.1 mmol/L before the administration of mulberry leaf powder. After continuous use of mulberry leaf powder for 10 weeks, although the blood glucose in the gavage group was still higher than that in the normal group, it was at a relatively low level compared with that in the diabetic control group. The glucose tolerance test (GTT) result indicated that mulberry leaf could enhance the insulin sensitivity of diabetic mice (Figure 2A; Table S2) and significantly reduce the fasting blood glucose (FBG) levels of diabetic mice after 4 weeks of treatment (Figure 2B; Table S3). In general, mulberry leaf could significantly reduce the FBG and improve the insulin sensitivity of diabetic mice.

**Figure 3:**
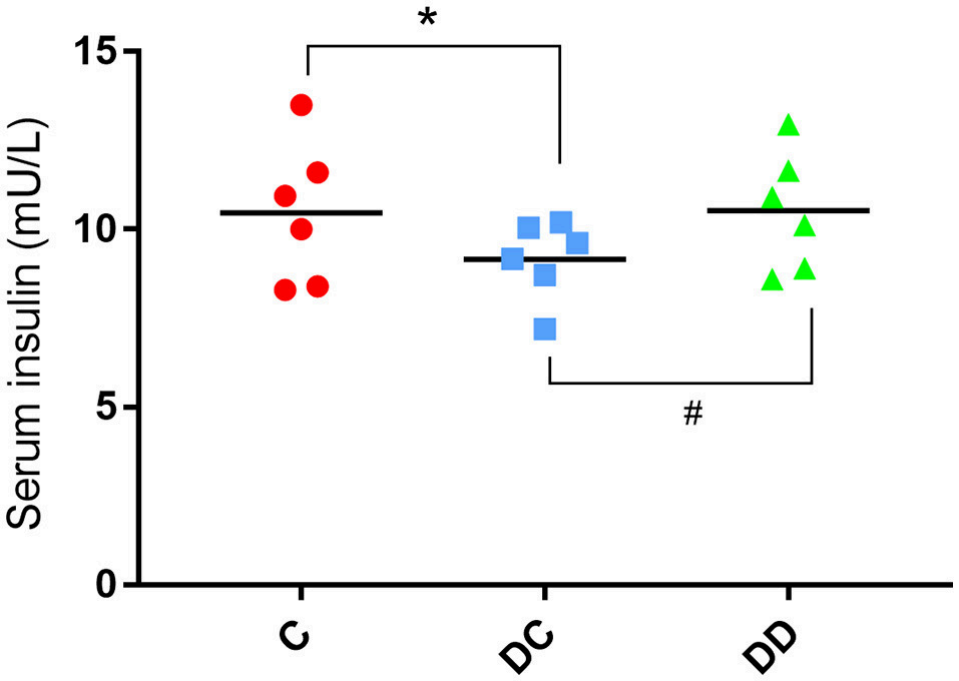
Serum insulin levels of the mice. The serum insulin levels in the STZ-induced diabetic mice were significantly lower than that in the normal mice (*P < 0.05, DC compared with C). The serum insulin levels in the mulberry powder-fed mice were significantly increased (^#^*P* < 0.05, DD compared with DC).

The results of the mouse insulin test showed that the serum insulin value in the diabetic model group before administration of mulberry leaf powder was slightly lower than that in the normal mice. After continuous use of mulberry leaf powder for 10 weeks, the levels of serum insulin in the diabetic control mice still showed a decreasing trend. The level of serum insulin in mulberry powder-fed mice was similar to that in normal mice (Figure 3; Table S4).

### Statistical analysis of raw data from mouse liver transcriptome

In order to obtain a broad, unbiased assessment of the diabetic process and mouse liver transcriptome kinetics after mulberry leaf powder treatment, gene transcription profiles were obtained by performing RNA sequencing for three libraries using the Illumina HiSeq (TM) 2500 system. The Q30 values in all three of the libraries were more than 90%, indicating a good alignment. The quality of the raw data was assessed prior to analysis using FASTQC (http://www.bioinformatics.babraham.ac.uk/projects/fastqc/), and low-quality fragments were removed by data preprocessing. From the libraries of the control, diabetic control, and diabetic mulberry-treated mice, 53,207,368, 53,253,970, and 53,253,770 high-quality reads were obtained, respectively, and subsequent studies of these high-quality reads were performed. The results of the treatment are shown in Tables 1, 2, and the average mass of the reads as a whole is very high, which shows that the theoretical value of the experiment is in good agreement with the measured value and the sequencing results are reliable.

**Table 1:** Before and after quality of pre-treatment data statistics.

**Sample**	**Raw reads**	**Raw bases**	**Clean reads**	**Clean bases**	**Valid ratio (base)**	**Q30 (%)**	**GC content (%)**
Sample_C	53,207,368	6,650,921,000	52,542,956	6,566,343,431	98.72%	97.04%	48.00%
Sample_DC	53,253,970	6,656,746,250	52,626,414	6,576,806,657	98.79%	97.11%	48.50%
Sample_DD	53,253,770	6,656,721,250	52,780,196	6,596,137,284	99.08%	97.38%	49.00%

*The statistical data here are the results of read1 + read2. C: normal mouse liver tissue; DC: liver tissue of the diabetic mice; DD: liver tissue of the diabetic treated mice. Raw reads: the total number of raw reads was counted in each sample; Raw bases: the total number of bases was counted in each sample sequenced; Clean reads: the calculation method was the same as that of raw reads, except the statistical files were the filtered sequencing data. The subsequent bioinformatics analysis was based on clean reads. Clean bases: the total number of bases was counted in the clean reads, and the results were translated to values in G; Q30: the number of bases in the original data was calculated with Phred values greater than 30% GC content: the percentage of the total number of bases G and C in the original data was calculated as a percentage of the total number of bases*.

**Table 2:** Distribution statistics of gene abundance (FPKM).

**Sample**	**Min**.	**1st Qu**.	**Median**	**Mean**	**3rd Qu**.	**Max**.	**Sum**.
Sample_C	0	0	0.2873965	16.3884005147551	4.356305	13,777.9	368,804.56518405
Sample_DC	0	0	0.3375105	16.8922774620034	4.23865	16,595.9	380,143.812004924
Sample_DD	0	0	0.2822355	17.1462552416959	4.18458	17,327.4	385,859.327959124

*FPKM: reads per 1 K bases of map to exons per 1 million map reads. Min, minimum; 1st Qu, first quartile; Median, median; Mean, average; 3rd Qu, third quartile; Max, maximum*.

### Differential gene expression changes in the three libraries

**Figure 4:**
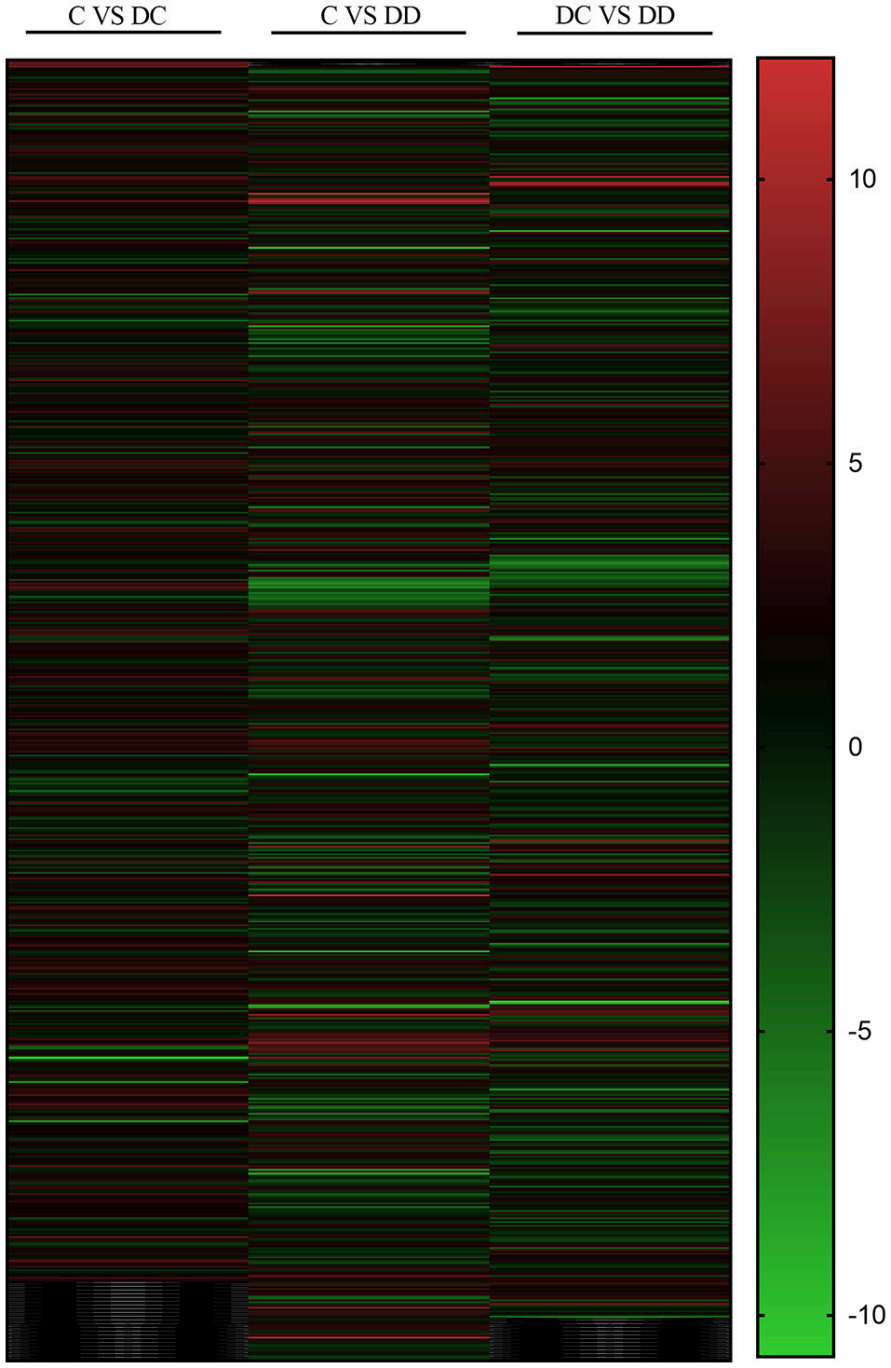
Differential gene change heat map. Red represents the upregulated genes, green represents the downregulated genes, and black represents no significant difference in genes.

The changes in DEGs are shown in Figure 4. The red field shows the upregulation of genes (absolute difference), while the green field shows the downregulation of genes. There were 652 common genes in the DC group (diabetic group) and C group (normal group), 694 common genes in the C group (normal group) and DD group (treatment group), and 671 common genes in the DC group (diabetic group) and DD group (treatment group). This suggests that the expression of many DEGs may be related to the recovery of STZ-induced liver injury after mulberry leaf powder treatment.

**Figure 5:**
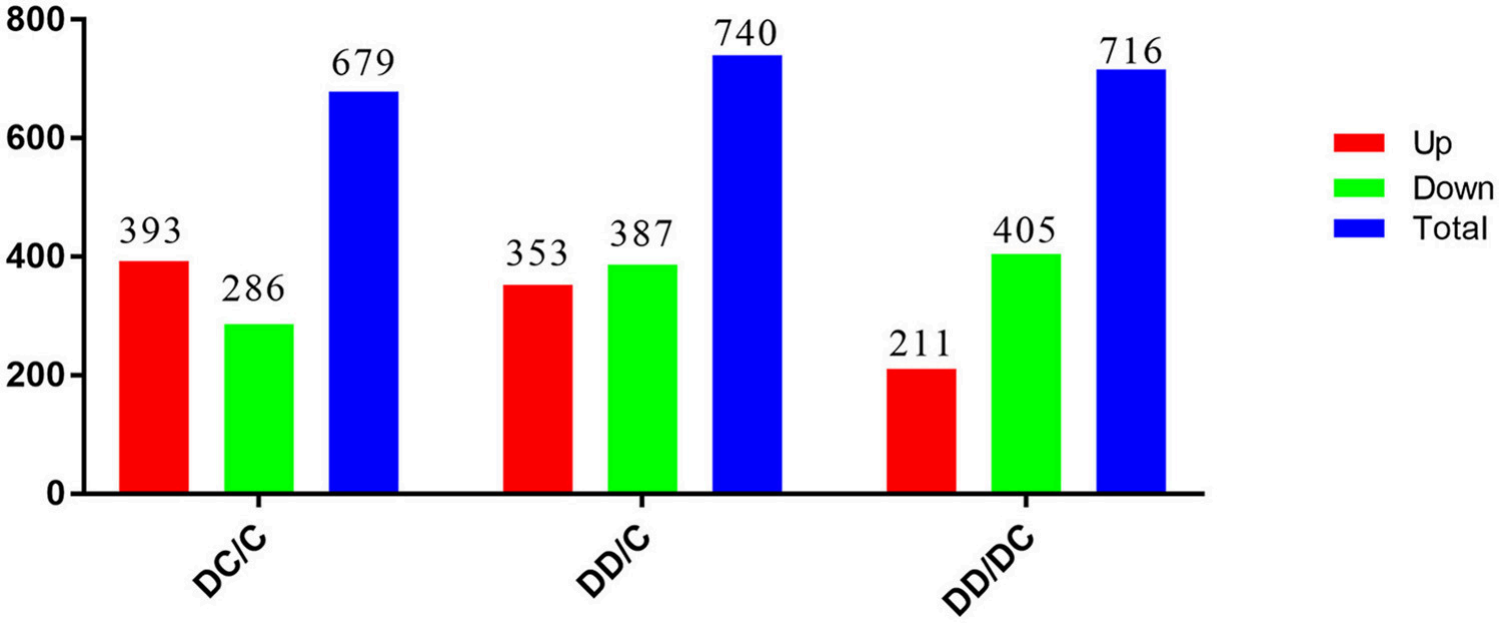
Differentially expressed gene statistics.

The uniquely expressed genes in the library should indicate a close relationship with liver damage, insulin signaling, inflammation, and glucose metabolism. Among them, the total number of DEGs of DC/C and DD/C were 679 and 740, respectively, of which 393 and 353 were upregulated and 286 and 387 were downregulated, respectively. However, comparing DD/DC, it was found that the total number of DEGs was 716, of which the number of upregulated genes was 211 and the number of downregulated genes was 405 (Figure 5).

**Figure 6:**
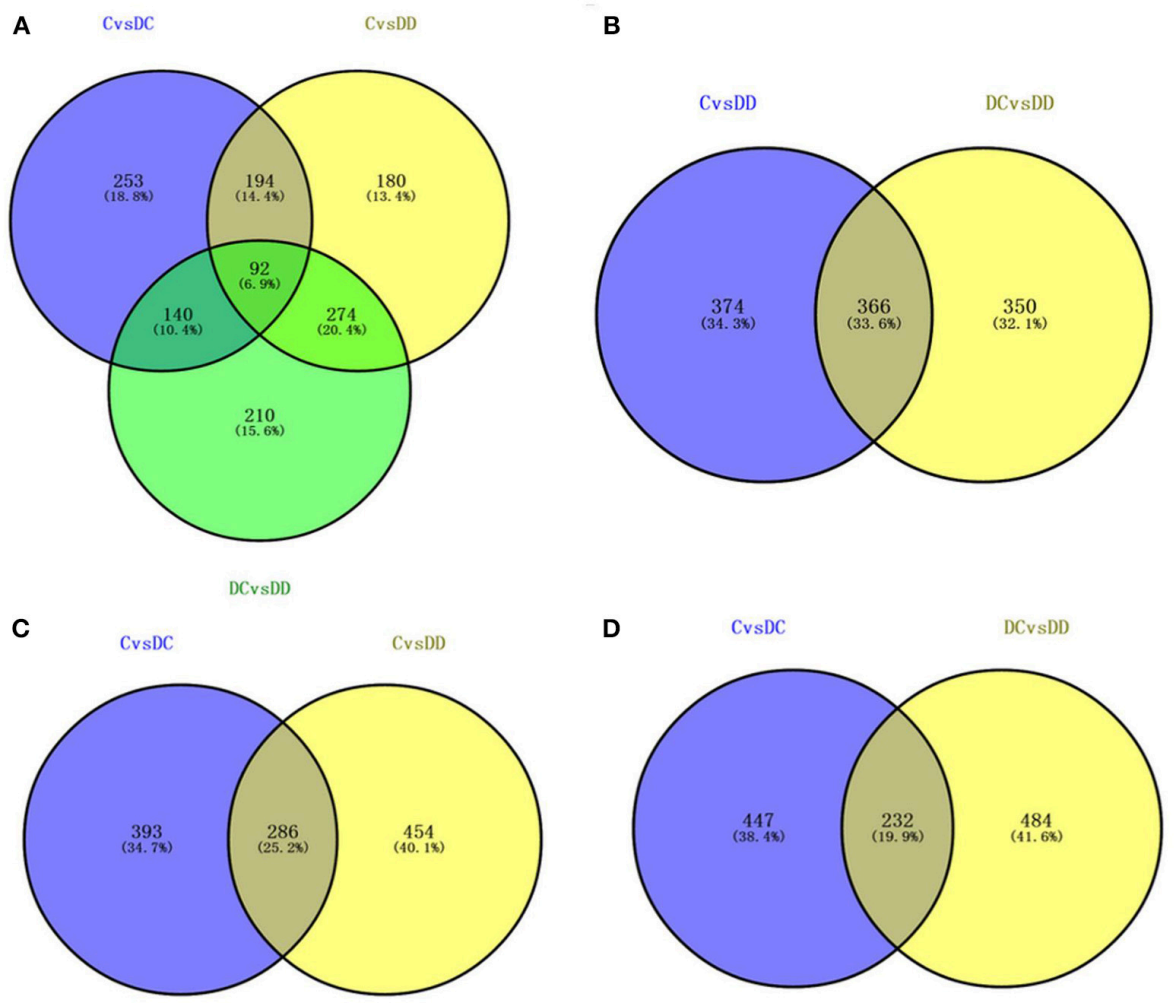
Differential gene expression by Venn diagram analysis. **(A)** Number of differential gene expression profiles in the three libraries. **(B)** The number of DEGs that overlap between C-DC and DC-DD. **(C)** The number of DEGs that overlap between C-DC and C-DD. **(D)** The number of overlapping DEGs between C-DC and DC-DD.

In order to study the molecular differences between the onset of diabetes and the stages of treatment with mulberry leaf powder, it is necessary to identify the DEGs in all three of the libraries. We identified genes with fold change ≥2 and FDR < 0.05 as significant DEGs. Venny online software (http://bioinfogp.cnb.csic.es/tools/venny/index.html) was used to obtain the intersection of DC/C, DD/C, and DD/DC, and determine the gene expression by analysis between the intersection and union (Figure 6A). The results showed that there were 92 DEGs in the three groups, 253 specific genes in DC/C, 180 specific genes in DD/C, and 210 specific genes in DD/DC. DEGs between DD/C and DD/DC were analyzed for intersection and union (Figure 6B), and the results showed that there were 366 DEGs, 374 genes specifically expressed in DD/C, and 350 genes specifically expressed in DD/DC. Analysis of the intersection and union of DEGs between DC/C and DD/C is shown in Figure 6C. The results showed that 286 DEGs were expressed in both groups, 393 were expressed in DC/C, and 454 were expressed in DD/C. DEGs between DC/C and DD/DC were analyzed for intersection and union (Figure 6D). The results showed that 232 DEGs were expressed in both groups, 447 were expressed in DC/C, and 484 were expressed in DD/DC.

### GO functional classification of DGEs

**Figure 7:**
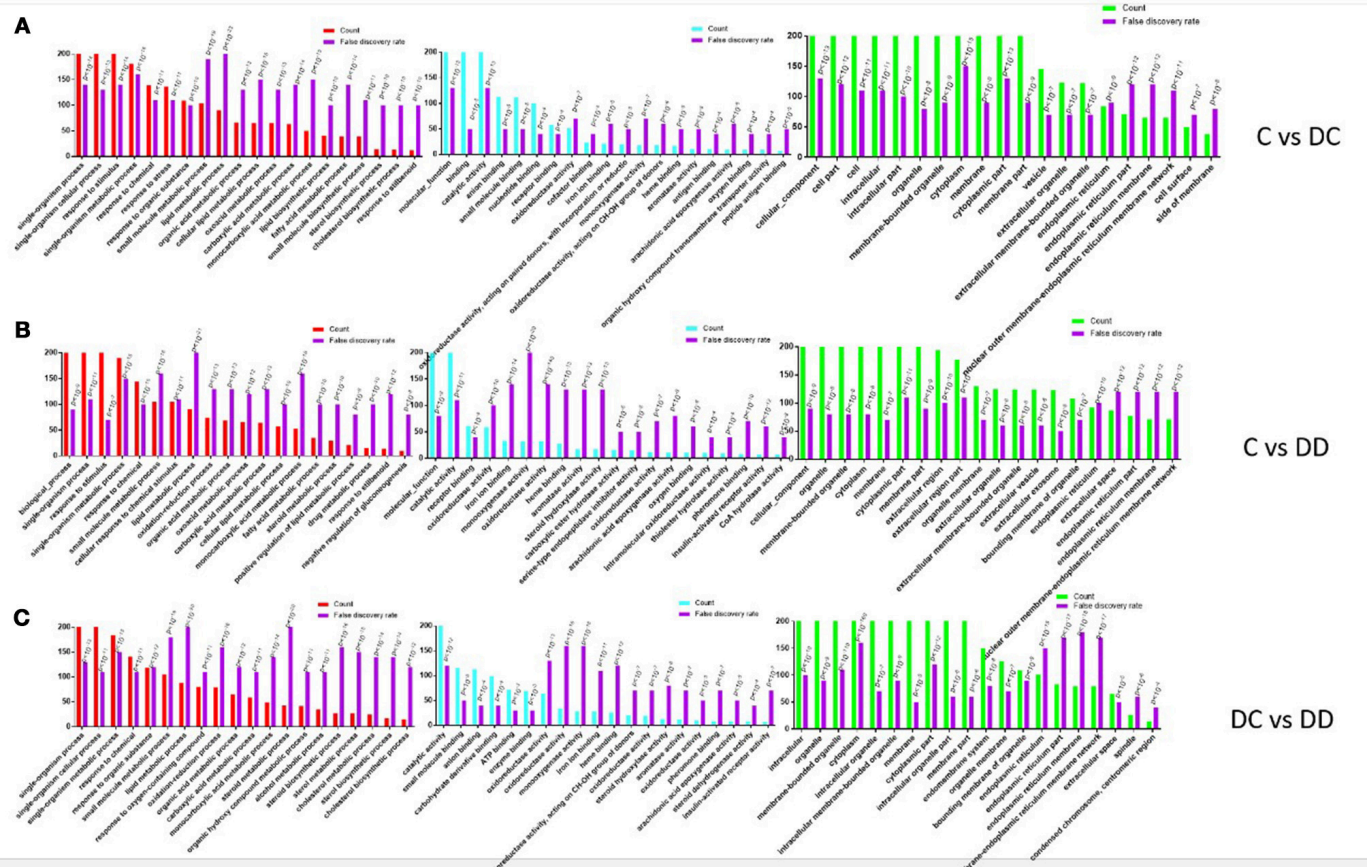
GO annotation of differentially expressed gene (DEG) functional classification. **(A)** GO analysis of DEGs of C-DC; **(B)** GO analysis of DEGs of C-DD; **(C)** GO analysis of DEGs of DC-DD.

After obtaining the DEGs, the top 20 of GO enrichment analysis were selected (according to the –log10 *P*-value order of each entry, and the entry containing less than three differential genes was filtered out). The DEGs were selected for GO enrichment analysis, and their functions were described in combination with GO annotation results. The standard GO classification provides a better understanding of the biological function of DEGs in the pathogenesis of diabetes and after mulberry leaf treatment. The DEGs of GO classification can be divided into three basic functional categories: Biological Process, Molecular Function, and Cellular Component. Three categories comparing the treatment groups (C-DC, C-DD, and DC-DD) are shown in Figure 7.

Most of the DEGs in the C-DC group were assigned to a single tissue process (377 genes, approximately 17% of DEGs), single cell processes (347 genes, approximately 15% of DEGs), and the stress response (246 genes, approximately 11% of DEG); most of the DEGs were classified into molecular functions (409 genes, approximately 26% of DEGs), binding (322 genes, approximately 20% of DEGs), and catalytic activity (228 genes, approximately 14% of DEGs); and in the cellular component category, cellular to cellular components (493 genes, approximately 10% of DEGs), cell composition (447 genes, approximately 9% of DEGs), and cells (447 genes, approximately 9% of DEGs). For the DEGs in the C-DD group, most were assigned to the biological processes (448 genes, approximately 20% of DEGs), single cell processes (392 genes, approximately 17% of DEGs), and stress response (236 genes, accounting for approximately 10% of DEGs). In the molecular functional category, most of the DEGs were classified into molecular functions (416 genes, accounting for approximately 40% DEGs), catalytic activity (237 genes, approximately 22% DEGs), and receptor binding function (61 genes, approximately 5% DEGs); in the cellular component class, the DEGs were classified into cellular components (517 genes, approximately 12% of DEGs), organellar composition (414 genes, approximately 10% of DEGs), and membrane bound (394 genes, approximately 9% of DEGs). For the DEGs in the DC-DD group, most were assigned to a single tissue process (389 genes, approximately 20% of DEGs), a single cellular process (352 genes, approximately 18% of DEGs), and single tissue metabolism (184 genes, accounting for approximately 9% of DEG); most of the DEGs were classified into molecular functions (401 genes, approximately accounting for 25% of DEGs), binding (323 genes, approximately 20% of DEGs), and catalytic activity (233 genes, approximately 14% of DEGs); in the cellular component category, the DEGs were grouped into cell composition (510 genes, approximately 9% of DEGs), cellular composition (462 genes, approximately 8% of DEGs), and cellular fractional composition (461 genes, approximately 8% of DEGs). The classification results showed that these DEGs comprising the key genes involved in enzyme-catalyzed functions during the pathogenesis of diabetes mellitus provide valuable information for further study on the pathogenesis of diabetes.

### KEGG classification of DGEs

**Figure 8:**
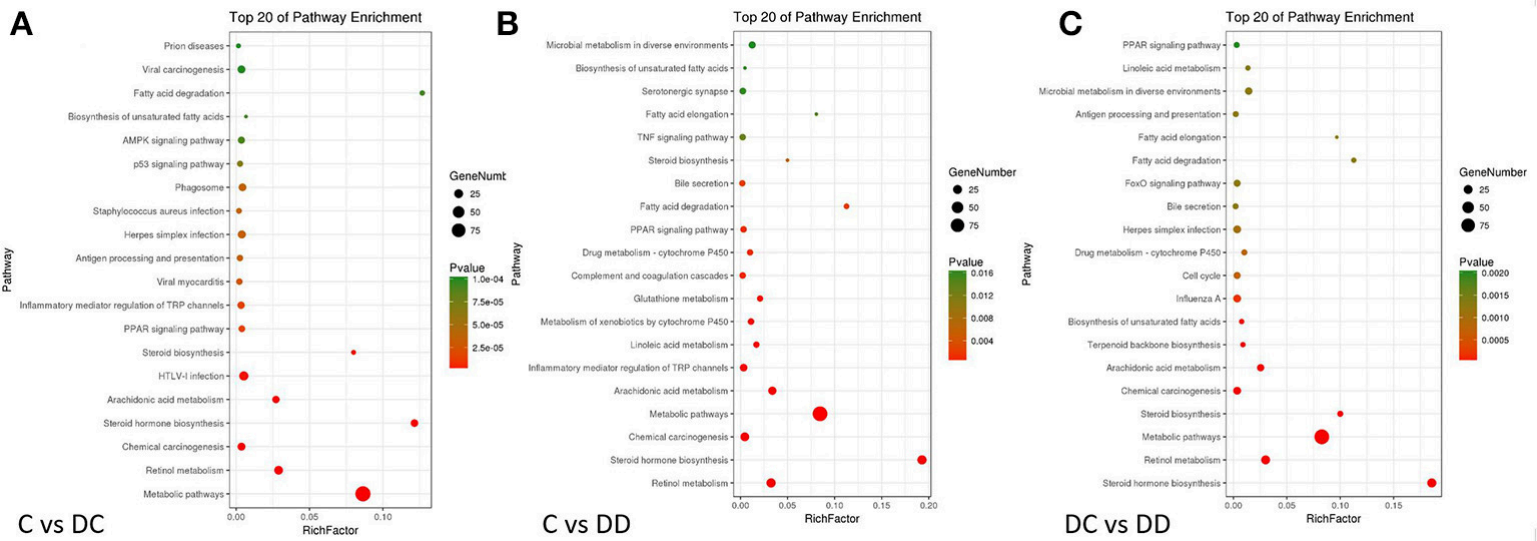
Bubble diagram of DGEs (differentially expressed genes) KEGG pathway analysis. **(A)** KEGG analysis of DGEs in C-DC; **(B)** KEGG analysis of DGEs in C-DD; and **(C)** KEGG analysis of DGEs in DC-DD. The enrichment score was calculated as: enrichment score = (*m*/*n*)/(*M*/*N*). Where *N* is the number of all genes with KEGG annotation, *n* is the number of DGEs in *N, M* is the number of all genes annotated to specific pathways, and *m* is the number of DGEs in *M*. Pathways with a *P*-value ≤ 0.05 were considered significantly enriched.

To identify the pathogenesis of diabetes and the biological pathway activated during mulberry leaf treatment, the three groups of DEGs were mapped into the KEGG database record of the pathway. Three groups of DEGs from the KEGG analysis FDR (False Discovery Rate) were selected. The top 20 pathways with the lowest FDR are shown by a bubble chart in Figure 8. For the C-DC group, 58 DEGs were involved in a total of 58 known KEGG pathways, many of which were classified into metabolic pathways, retinol metabolism, inflammatory mediators of TRP channels, PPAR signaling, AMPK signaling pathway, fatty acid degradation and biosynthesis pathway, insulin signaling pathway, and glucose metabolism/gluconeogenesis pathway. DEGs in the C-DD group were involved in 24 known KEGG pathways, of which many DEGs were involved in steroid biosynthesis, retinol metabolism, metabolic pathway, drug metabolism-cytochrome P450, PPAR signaling pathway, fatty acid degradation, TNF signaling pathway, microbial metabolism in different environments, and AMPK signaling pathway. There were 37 known KEGG pathways in the DC-DD group, of which many DEGs involved steroid hormone biosynthesis, retinol metabolism, metabolic pathway, drug metabolism-cytochrome P450, fatty acid degradation and biosynthesis pathway, PPAR signaling pathway, inflammatory mediators of TRP channel, tyrosine metabolism, AMPK signaling pathway, and adipocytokine signaling pathway. The KEGG classification of DEGs showed that most metabolic pathways were closely related to glucose metabolism, insulin signaling, and inflammation.

### Transcriptional level verification of DGEs

**Figure 9:**
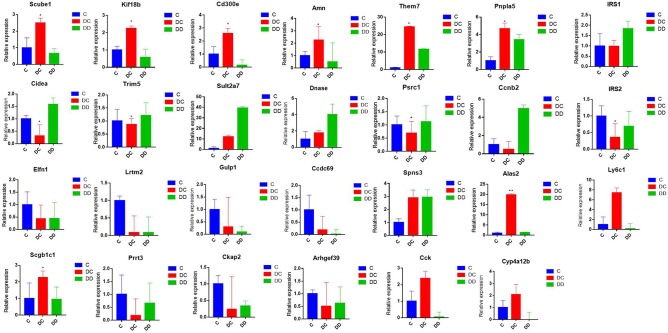
Validation of the expression pattern of DGEs associated with diabetes by qRT-PCR; qRT-PCR used the same RNA samples (three mixed samples from each treatment group). All of the qRT-PCR data are shown as the mean ± standard error; *n* = 3. Their expression relative to β-actin was quantified by qRT-PCR.

In order to verify the accuracy of the Illumina sequencing, we selected 27 DEGs (Scube1, Kif18b, Cd300e, Amn, Them7, Pnpla5, Cidea, Trim5, Sult2a7, DNase, Psrc1, Ccnb2, Elfn1, Lrtm2, Gulp1, Ccdc69, Spns3, UBD, Scgb1c1, Prrt3, Ckap2, Arhgef39, Cck, Cyp4a12b, IRS1, IRS2, and Ly6c1) that were used to verify the differences obtained from transcriptome sequencing by qRT-PCR. The results indicate that the gene expression profiles of these DEGs verified by qRT-PCR revealed similar trends when compared to the RNA-Seq samples (Figure 9; Table S6), indicating the high confidence of the transcriptional abundance of these DEGs in signaling.

### Western blot validates differentially expressed proteins

**Figure 10:**
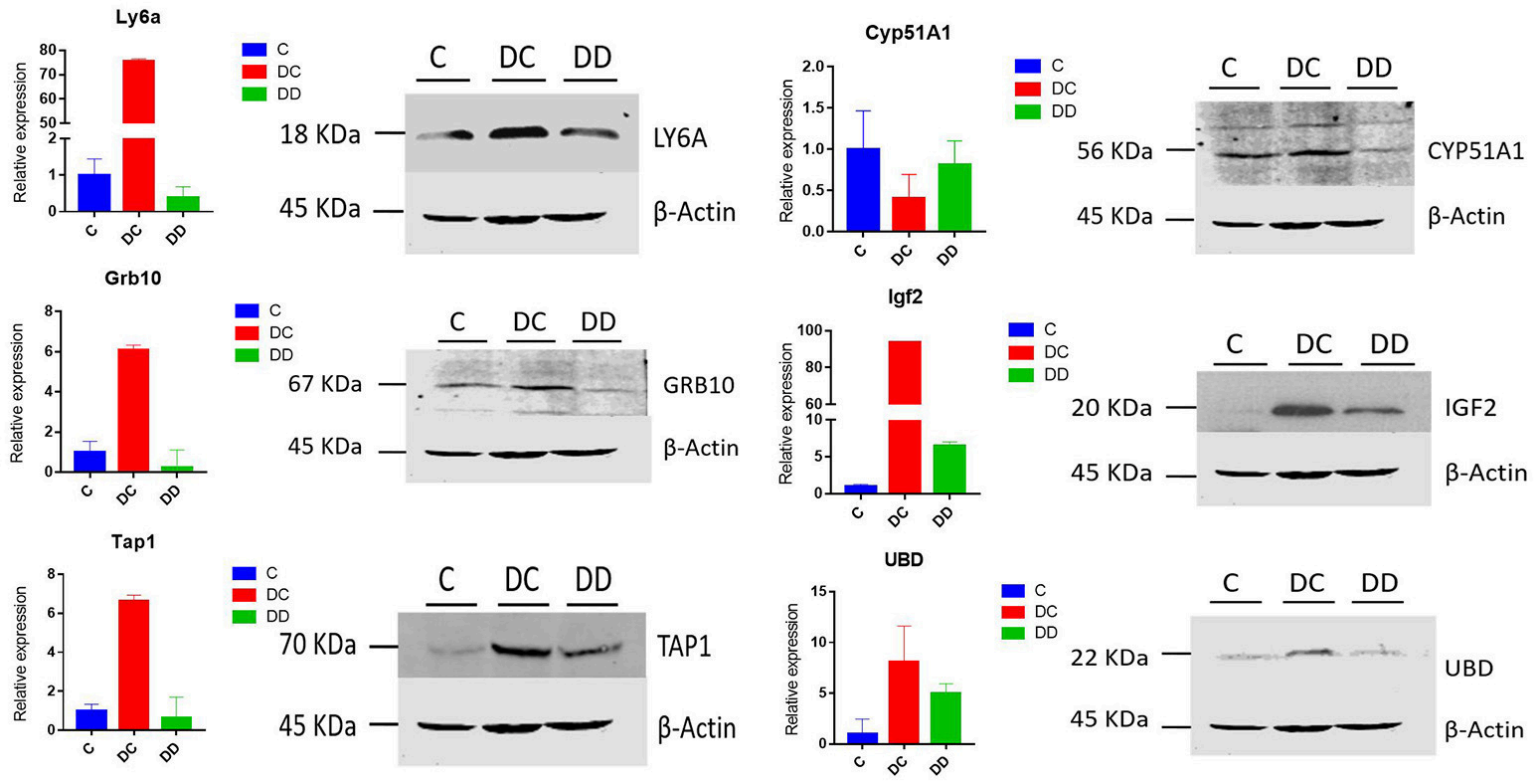
The expression patterns of differentially expressed genes associated with the onset of diabetes are shown; expression was determined by qRT-PCR and Western blot, with RNA samples and protein positives from three mixed samples per treatment group. All of the qRT-PCR data are shown as the mean ± standard error; *n* = 3. Their expression relative to β-actin was quantified by qRT-PCR.

Western blotting was used to further confirm the changes in the differential protein expression in mouse livers from diabetic and mulberry leaf-treated mice. LY6A, CYP51A1, IGF*2*, TAP1, GRB10, and UBD proteins were selected for analysis, and β-actin was used as a control. Compared with group C, the expression of LY6A, IGF2, TAP1, Grb10, and UBD was upregulated, and the expression of CYP51A1 was downregulated in the DC group. The expression of IGF2 and UBD was upregulated, and the expression of LY6A, CYP51A1, TAP1, and Grb10 was downregulated in the DD group (Figure 10). For our validation of these six proteins, the Western blot results were in good agreement with our RNA-Seq transcriptome sequencing and qRT-PCR analysis (Figure 10). The changes in the transcriptional level and protein level of Ly6a, Grb10, Tap1, IGF2, and UBD in the livers of mice from the three groups were both significantly expressed in the DC group and suppressed in the DD group, while the change in CYP51A1 expression was inhibited in the DC group and significantly enhanced in the DD group.

### Effect of active ingredients in mulberry leaf powder on differentially expressed proteins in the pathogenesis of diabetes mellitus

**Figure 11:**
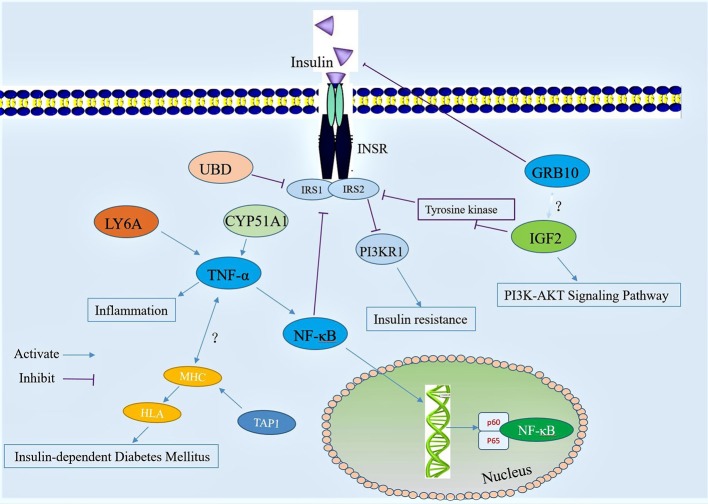
Diagram illustrating a hypothetical mechanism of action for the differential expression of protein that occurred after treatment with mulberry leaf powder. The red circles represent the active compound in the mulberry leaf component.

The above test results analyzed the dynamic changes of the differentially expressed proteins in diabetic mice after mulberry leaf powder treatment, and combined with other research results regarding the differential proteins, we propose a mechanism of action for the active compound in mulberry leaf powder (Figure 11), as well as for the differential expression of proteins caused by the disorder of glucose metabolism pathways and inflammatory signaling. According to a study by Sia et al. (Sia and Weinem, 2005), the overexpression of the *Tap1* gene leads to incorrect antigen processing, disruption of self-peptide presentation, and reduction of cell surface expression of MHC class 1 molecules. The genetic control of insulin-dependent diabetes mellitus (IDDM) mainly depends on the *HLA* gene in the MHC, while the *TAP1* gene is involved in its regulation (Caillat-Zucman et al., 1992), andTT *TAP1* may induce insulin-dependent diabetes mellitus. It has also been previously reported that TNF-α may play an independent role in the pathogenesis of IDDM (Yu et al., 1999).

Ubiquitin D (UBD) expression is upregulated in the pathogenesis of diabetes, and UBD inhibits the activity of insulin receptor substrates 1 and 2 (IRS1 and IRS2), diminishing their ability to accept insulin, which leads to the downregulation of PI3KR1 expression and causes insulin resistance. The upregulation of lymphocyte antigen 6A (LY6A) and the downregulation of CYP51A1 can activate the expression of TNF-α and NF-κB, leading to inflammation. The growth factor overexpression of Grb10 can block the signal between insulin and insulin receptor, inducing insulin resistance. Insulin-like growth factor II (IGF-2) upregulates the activity of IRS1 and IRS2 by activating the expression of tyrosinase and it can also affect the PI3K-AKT signaling pathway, resulting in the disorder of glycometabolism. The active compounds in mulberry leaves regulate the expression of these different proteins to ease the incidence of diabetes. In addition, these differentially expressed proteins may also cause blocking of other signaling pathways, which requires further study. In conclusion, the preliminary mapping provided by our trial results will help us to understand the mechanism of mulberry leaf powder in the treatment of diabetes.

## Discussion

A healthy diet is an essential factor to lower the risk of DM development. Considering adverse effects of drugs, natural products from traditional Chinese medicine like Mulberry leaves have been drawn extensive attentions on preventive and therapeutic interventions for DM (Yan et al., 2016). Using network pharmacological analysis, Ge et al. found that components of mulberry leaves can relieve the symptoms of diabetes through the action of target proteins in various metabolic pathways (Ge et al., 2018). However, the active compound with hypoglycemic activity of mulberry leaves are still unknown.

Among them, 1-deoxynojirimycin (1-DNJ) is a polyhydroxy alkaloid derived from mulberry leaves (Huang et al., 2014). As an analog of D-glucose, it has potent α-glycosidase inhibitory activity and effectively reduces postprandial blood glucose levels. A large number of scientific experimental results (Ji et al., 2016) have shown that the hypoglycemic effect of mulberry leaves can be achieved through the following two ways: (1) DNJ substances contained in mulberry leaves prevent the production of new glucose by inhibiting the activity of disaccharidases; and (2) the fagomine alkaloid in mulberry leaves stimulates the beta cells to secrete insulin so that the glucose in the blood is metabolized, and the synthesis of glycogen is promoted at the same time, finally achieving the purpose of lowering blood sugar (Hunyadi et al., 2013). Numerous clinical trials have shown that DNJ strongly inhibits the disaccharidase activity inside the human digestive tract, such as that of sucrase or isomaltose enzyme, and it also reduces the possibility of the conversion of disaccharides in the body to glucose, but it has no effect on sugar absorption at the same time. The hypoglycemic mechanism of fagomine in mulberry leaves involving the increase of insulin release is similar to that of glibenclamide, which is generally believed to play a role in the small intestine after oral administration and delay the absorption of carbohydrates in food, thus achieving the effect of lowering blood glucose (Hunyadi et al., 2013). Clinical studies have shown that DNJ can inhibit the absorption of sucrase, maltase, isomaltase, trehalase, and lactase by small intestine microvilli and it has the strongest inhibitory effect on sucrase, but it does not inhibit α-amylase or cause sugar absorption disorders. Therefore, the main goal of diabetes treatment is to maintain normal blood glucose levels. During the treatment of type II diabetes, DNJ can be used alone or in combination with insulin secretion or sensitizing agents depending on the cause and existing metabolic disorders. Another function of α-glucosidase inhibitors is to reduce carbohydrate absorption in the intestine and lower blood glucose levels (Król et al., 2016).

In this experiment, a single intraperitoneal injection of STZ induced diabetes in mice, which resulted in high blood sugar, insulin resistance, and other symptoms of diabetes. The amount of blood sugar in these mice sharply increased and serum insulin decreased 72 h after the injection. After 10 weeks of administration to diabetic mice, the mulberry leaf powder had a positive effect on fasting blood glucose and insulin levels. After mulberry leaf administration, diabetic mice experienced a significant increase in body weight compared to the DC group (Figure 1). It has also been reported (Pelantová et al., 2016) that after a certain period of treatment, body weight decreased but the other physiological indexes were significantly different. Yet another study (Liu et al., 2015) reported no significant difference during the first week of treatment, with DNJ-treated diabetic mice exhibiting a significantly reduced body weight from the second week in a dose-dependent manner. Do et al. (2015) concluded that the final body weight of the high-fat group (38.08 g) was significantly higher than that of the control group (30.00 g) or DNJ group (34.01 g).

Our results are consistent with previous findings (Aramwit et al., 2013), although our treatment span of 10 weeks may be longer than other studies. Therefore, on the basis of comparing the results of previous studies, we can conclude that the aqueous solution of mulberry leaf powder has little effect on weight regulation in mice. Previously, Reed et al. (2000) injected normal rats with STZ to induce the development of diabetes mellitus. Compared with the fasting blood glucose of normal rats, the fasting blood glucose of STZ-injected rats was significantly increased, and decreased pancreatic β-cell function and increased insulin resistance were observed in STZ-induced diabetic rats. Thus, STZ can be used to produce a hyperglycemic animal model with the administration of a single high dose, and type II or type I diabetic animal models can also be produced using a variety of low-dose STZ treatments. In addition, mulberry leaf powder application significantly improved glucose tolerance in diabetic mice, indicating that the construction of a diabetic mouse model basically meets the experimental requirements. In this experiment, feeding mulberry leaf water solution (80 mg/kg) to diabetic mice significantly reduced fasting blood glucose (FBG) and increased serum insulin levels, resulting in reduced insulin resistance and improved insulin receptor sensitivity. Mulberry leaf as a traditional Chinese medicine treating diabetes mainly by enhancing the insulin sensitivity of peripheral tissues.

Previously published studies (Aramwit et al., 2011) reported that mulberry aqueous solution can inhibit TNF-α-induced NF-κB activation and Lectin-like Oxidized Low Density Lipoprotein Receptor-1 expression in vascular endothelial cells. Asai et al. (2011) showed that long-term intake of mulberry leaf extract enriched in DNJ can improve postprandial glucose levels in individuals with impaired glucose metabolism. Toshiyuki et al. (2007) found that mulberry aqueous ethanol extract effectively reduced postprandial blood glucose and increased insulin concentrations in healthy subjects. Sheng et al. (2017) showed that mulberry leaf is effectively used in TCM for the treatment of diabetes. Mulberry leaf is effective due to its mechanism of action, which involves attenuating NEFA (non-esterified fatty acid) signaling as well as regulating gut microbiota in STZ-induced diabetic rats, stimulating the release of insulin to enhance insulin sensitivity in peripheral tissues (Beck-Nielsen et al., 1979), and inhibiting glycogenolysis and gluconeogenesis (Carvalho-Martini et al., 2006).

Hamdy et al. (Hamdy, 2012) reported that mulberry leaf extract given to type II diabetic rats can reduce blood glucose levels, regulate oxidative stress levels, increase hexokinase and glycogen synthesis activity, and reduce glucose metabolism in the liver via a reduction of glucose-6-phosphatase activity. Andallu et al. (2001) found that oral administration of mulberry leaf extract reduced blood glucose, triglycerides, very low density lipoprotein (VLDL) cholesterol, low density lipoprotein (LDL) cholesterol, and fatty acids in patients with type II diabetes. Aramwit et al. (2011) also found that mulberry leaf powder can effectively reduce blood lipids in patients with mild hyperlipidemia, and mulberry leaf treatment more effectively inhibits triglycerides and LDL than dietary control. From this, it can be seen that the anti-diabetic mechanism of mulberry leaves appears to be multidirectional, of which the most effective is DNJ, a nitrosyl sugar that mainly inhibits alpha-amylase and galactosidase (Hunyadi et al., 2012), while the polysaccharide is an α-glucosidase competitor. In addition, mulberry leaves contain phenolic substances with strong antioxidative capacity to reduce oxidative stress and related complications of diabetes (Katsube et al., 2006).

In the current study, diabetic mice exhibited constant hyperglycemia during the entire experiment compared to normal mice. After 10 weeks of mulberry leaf powder administration, FBG was significantly reduced compared with the diabetic group and the control group (Figure 2). Previous studies have also reported a significant reduction in FBG in diabetic mice following antidiabetic drug administration (Toshiyuki et al., 2007). Serum insulin was monitored 10 weeks after treatment with mulberry leaf powder. The average insulin level in diabetic mice was lower than that in the normal and treatment groups (1.23 and 1.28 μg/L, respectively) (Figure 3). Lack of early insulin secretion is a classic deficiency of type II diabetes and may lead to high FBG concentrations and postprandial hyperglycemia (Yi et al., 2012). Therefore, our results are basically consistent with the reduction in serum insulin levels as previously reported with other diabetic model mice (Shibata et al., 2007).

The pathogenesis of diabetes is a complex process involving the regulation of many genes. In order to identify the process of diabetes mellitus and the DEGs after mulberry leaf powder treatment, the liver tissues of normal mice, diabetic mice, and mulberry leaf powder-treated diabetic mice were collected and sequenced. According to the gene expression analysis, there were significant DEGs among the groups. In this experiment, we measured gene transcripts in the liver from the three groups of mice, focusing on genes involved in the balance of glucose metabolism, inflammation, and insulin signaling. The results showed that there was a significant difference between the diabetic group and the normal group and the mulberry group. Compared with the normal group, mulberry leaves significantly restored the transcription level of these genes. We also selected more significant DEGs for qRT-PCR to verify the transcriptional level of these genes and transcriptome sequencing differences. Here, we selected 27 DEGs that may participate in glucose metabolism and are related to inflammation. According to our analysis, most DEGs were significantly upregulated when compared to diabetic groups and normal groups. After comparing the control mice with the mice that were treated with mulberry leaves, we found that most of the significantly DEGs gradually recovered to normal levels of expression. Based on these findings, we have suggested that changes in the mRNA levels that lead to these DEGs may be due to the restoration of gene expression caused by the treatment with mulberry leaves. This is consistent with a previous study by Rubin et al. (2016) showing that the expression of DEGs in diabetic nephropathy mice returned to normal levels after pigment epithelium-derived factor (PEDF) peptide treatment.

The ubiquitinated protein (UBD) downregulates the insulin receptor substrate protein (IRS2) in differentially expressed proteins validated by Western blot, leading to a decrease in the sensitivity of the body to insulin, which can result in the development of diabetes (Cort et al., 2014). Insulin-like growth factor 2 (IGF2) can activate tyrosine kinase upregulation by binding to IGF1, and it also leads to a decrease in the activity of insulin receptor substrate (IRS1), which can result in the development of diabetes (Su et al., 2016). IGF2, produced and secreted by adult β-cells, also acts as an autocrine activator of the β-cell insulin-like growth factor receptor signaling pathway and it is also capable of activating the expression of IGF-IR and AKT kinases. In addition, the overexpression of IGF2 leads to the de-differentiation of β-cells *in vivo* and pancreatic islet dysfunction caused by endoplasmic reticulum stress, making the islets more likely to initiate β-cell damage and immune attack. Casellas et al. (2015) showed that islet insulin-like growth factor (IGF2) may lead to the development of diabetes mellitus. Further experiments by Mu et al. (2015) showed that IGF2 activity is upregulated and can activate PI3K/AKT signaling. Besides, Ong et al. found that either genetic or pharmacological activation of Akt protected the heart against acute ischaemia-reperfusion injury by modulating mitochondrial morphology (Ong et al., 2015). Our validation of IGF2 showed that it is highly expressed in the liver of diabetic mice, which is consistent with previous studies. Thus, IGF2 is an autocrine ligand of the β-cell IGF1R receptor, and GLP-1 enhances the autocrine activity by enhancing the expression of IGF-1R, promoting the transcription of endothelial growth factor (EGFR), and activating phosphoinositide kinase (PI3K) (Honey et al., 2014).

Stem cell antigen 6 complex (LY6A) induces the expression of interleukins, such as lymphokines, TNF-α, IL-6 and IL-9, resulting in the production of inflammatory signals (Chen et al., 2003); growth factor receptor binding protein 10 (Grb10) binds to and inhibits the activated receptor tyrosine kinase and simultaneously inhibits insulin binding to insulin-like growth factor (IGF-1), which interferes with signal transduction and increases receptor degradation. Blocking the association of insulin and insulin receptor substrates 1 and 2 prevents their tyrosine phosphorylation. Research by Yang et al. (2016) showed that Grb10 expression was significantly elevated in the kidneys of diabetic mice compared to non-diabetic mice, whereas treatment with catalpol significantly abolished the increase in Grb10 expression in diabetic kidneys. In addition, a study by Ren et al. (2016) found that CYP51A1 (lanosterol 14-α-demethylase) is involved in the metabolism of sulfonylureas in type II diabetes and can cause changes in the amount of glycosylated hemoglobin (HbA1c); Ding et al. (2015) found that CYP51A1 is involved in cholesterol metabolism. In the current study, the expression of CYP51A1 was inhibited, which will cause the upregulation of the NF-κB signaling pathway, leading to the upregulation of TNF-α, which will then trigger an inflammatory reaction and aggravate the symptoms of diabetes.

It is well-known that the most important reason for the development of diabetes is that insulin resistance leads to impaired insulin secretion due to the impaired insulin signaling pathway, which leads to hyperglycemia. The insulin receptor is also a tyrosine kinase receptor that mediates the pleiotropic effects of insulin (Liu et al., 2016). Insulin binding leads to the phosphorylation of several intracellular substrates, including the insulin receptor substrate (IRS) (Fritsche et al., 2008). Insulin exerts its physiological functions through the insulin or insulin-like growth factor (IGF) signaling system. In this signaling system, insulin regulates metabolic processes, whereas insulin-like growth factors primarily promote cell division and differentiation. Insulin receptor substrate 2 (IRS2) plays a crucial role in the IN/IGF signaling system. The interaction between IGF2 and UBD causes a decrease in IRS expression, which results in the activation of the PI3K-AKT PKB pathway; this pathway is responsible for most of the metabolic effects of insulin and impairs the expression of certain genes. Therefore, the inactivation of IRS may lead to disorders in glucose and lipid metabolism and other effects caused by damage resulting from excess insulin.

## Conclusion

Based on RNA-Seq transcriptomics, we identified some differentially expressed genes involved in the pathogenesis of diabetes and elucidated the potential molecular mechanism caused by mulberry leaf powder in diabetic mice. The active ingredients in mulberry leaf powder exerted their effects by acting on UBD, IGF2, Grb10, and other target proteins, including the prevention of inactivation of inducible insulin receptor substrate (IRS), which normally would result in insulin resistance when inactivated. Finally, we present a hypothetical model for how mulberry leaf attenuates diabetes (Figure 11) that will help us to understand more about the molecular mechanisms of diabetes.

## Author contributions

QG and SZ are the major equal contributors in this manuscript. QG and SZ wrote the first draft of the manuscript and finalized the manuscript. LC, SM, and MT performed the statistical analysis. YY, PL, and QY processed the graph and the table in the manuscript. LL, MNK, LG, and MK revised sections of the manuscript. KC and FF contributed conception and design of the study. All authors contributed to manuscript revision and approved the submitted version of the manuscript.

### Conflict of interest statement

The authors declare that the research was conducted in the absence of any commercial or financial relationships that could be construed as a potential conflict of interest.

## References

[B1] Andallu B. Suryakantham V. Lakshmi S. B. Reddy G. K. (2001). Effect of mulberry (*Morus indica* L.) therapy on plasma and erythrocyte membrane lipids in patients with type 2 diabetes. Clin. Chim. Acta 314, 47–53. 10.1016/S0009-8981(01)00632-5 11718678

[B2] Ann J. Y. Eo H. Lim Y. (2015). Mulberry leaves (*Morus alba* L.) ameliorate obesity-induced hepatic lipogenesis, fibrosis, and oxidative stress in high-fat diet-fed mice. Genes Nutr. 10, 1–13. 10.1007/s12263-015-0495-x 26463593 PMC4604156

[B3] Aramwit P. Petcharat K. Supasyndh O. (2011). Efficacy of mulberry leaf tablets in patients with mild dyslipidemia. Phytother. Res. 25, 365–369. 10.1002/ptr.3270 20687135

[B4] Aramwit P. Supasyndh O. Siritienthong T. Bang N. (2013). Mulberry leaf reduces oxidation and c-reactive protein level in patients with mild dyslipidemia. BioMed Res. Int. 2013:787981. 10.1155/2013/787981 23484158 PMC3581086

[B5] Asai A. Nakagawa K. Higuchi O. Kimura T. Kojima Y. Kariya J.. (2011). Effect of mulberry leaf extract with enriched 1-deoxynojirimycin content on postprandial glycemic control in subjects with impaired glucose metabolism. J. Diabetes Investig. 2, 318. 10.1111/j.2040-1124.2011.00101.x 24843505 PMC4014974

[B6] Beck-Nielsen H. Pedersen O. Lindskov H. O. (1979). Increased insulin sensitivity and cellular insulin binding in obese diabetics following treatment with glibenclamide. Acta Endocrinol. 90, 451. 10.1530/acta.0.0900451 106617

[B7] Caillat-Zucman S. Bertin E. Timsit J. Boitard C. Assan R. Bach J. F. (1992). TAP1 and TAP2 transporter genes and predisposition to insulin dependent diabetes mellitus. C. R. Acad. Sci. III 315, 535–539. 1300236

[B8] Carvalho-Martini M. de Oliveira D. S. Suzukikemmelmeier F. Bracht A. (2006). The action of glibenclamide on glycogen catabolism and related parameters in the isolated perfused rat liver. Res. Commun. Mol. Pathol. Pharmacol. 119, 115–126. 17974101

[B9] Casellas A. Mallol C. Salavert A. Jimenez V. Garcia M. Agudo J.. (2015). Insulin-like growth factor 2 overexpression induces β-cell dysfunction and increases beta-cell susceptibility to damage. J. Biol. Chem. 290, 16772. 10.1074/jbc.M115.642041 25971976 PMC4505425

[B10] Chang C. Lin Y. Bartolome A. P. Chen Y. C. (2013). Herbal therapies for type 2 diabetes mellitus: chemistry, biology, and potential. Evid. Based Complement. Alternat. Med. 2013, 378657. 10.1155/2013/378657 23662132 PMC3638592

[B11] Chen H. C. Frissora F. Durbin J. E. Muthusamy N. (2003). Activation induced differential regulation of stem cell antigen-1 (Ly-6A/E) expression in murine B cells. Cell. Immunol. 225, 42–52. 10.1016/j.cellimm.2003.09.006 14643303

[B12] Cort L. Habib M. Eberwine R. A. Hessner M. J. Mordes J. P. Blankenhorn E. P. (2014). Diubiquitin (Ubd) is a susceptibility gene for virus-triggered autoimmune diabetes in rats. Genes Immun. 15, 168. 10.1038/gene.2013.72 24452267 PMC4260472

[B13] Davis P. A. Pagnin E. Semplicini A. Avogaro A. Calò L. A. (2006). Insulin signaling, glucose metabolism, and the angiotensin II signaling system: studies in Bartter's/Gitelman's syndromes. Diabetes Care 29, 469–471. 10.2337/diacare.29.02.06.dc05-2048 16443917

[B14] Ding J. Reynolds L. M. Zeller T. Müller C. Lohman K. Nicklas B. J.. (2015). Alterations of a cellular cholesterol metabolism network are a molecular feature of obesity-related type 2 diabetes and cardiovascular disease. Diabetes 64, 3464. 10.2337/db14-1314 26153245 PMC4587646

[B15] Do H. J. Chung J. H. Hwang J. W. Kim O. Y. Lee J. Y. Shin M. J. (2015). 1-Deoxynojirimycin isolated from *Bacillus subtilis* improves hepatic lipid metabolism and mitochondrial function in high-fat-fed mice. Food Chem. Toxicol. 75, 1. 10.1016/j.fct.2014.11.001 25445511

[B16] Fritsche L. Weigert C. Häring H. U. Lehmann R. (2008). How insulin receptor substrate proteins regulate the metabolic capacity of the liver–implications for health and disease. Curr. Med. Chem. 15, 1316–1329. 10.2174/092986708784534956 18537611

[B17] Gao Y. Liao G. Xiang C. Yang X. Cheng X. Ou Y. (2016). Effects of phycocyanin on INS-1 pancreatic β-cell mediated by PI3K/Akt/FoxO1 signaling pathway. Int. J. Biol. Macromol. 83, 185–194. 10.1016/j.ijbiomac.2015.11.054 26616456

[B18] Ge Q. Chen L. Chen K. (2017). Treatment of diabetes mellitus using iPS cells and spice polyphenols. J. Diabetes Res. 2017, 5837804. 10.1155/2017/5837804 28758131 PMC5512026

[B19] Ge Q. Chen L. Tang M. Zhang S. Liu L. Gao L.. (2018). Analysis of mulberry leaf components in the treatment of diabetes using network pharmacology. Eur. J. Pharmacol. 833, 50–62. 10.1016/j.ejphar.2018.05.021 29782863

[B20] Hamdy S. M. (2012). Effect of *Morus alba* Linn extract on enzymatic activities in diabetic rats. J. Appl. Sci. Res. 8, 10–16.

[B21] Honey M. Marion C. Bernard T. (2014). Glutamine stimulates biosynthesis and secretion of insulin-like growth factor 2 (IGF2), an autocrine regulator of beta cell mass and function. J. Biol. Chem. 289, 31972–31982. 10.1074/jbc.M114.587733 25271169 PMC4231675

[B22] Hu X. Q. Thakur K. Chen G. H. Hu F. Zhang J. G. Zhang H. B. (2017). Metabolic effect of 1-deoxynojirimycin from mulberry leaves on db/db diabetic mice using LC–MS based metabolomics. J. Agric. Food Chem. 65, 4658–4667. 10.1021/acs.jafc.7b01766 28541040

[B23] Huang S. S. Yan Y. H. Ko C. H. Chen K. M. Lee S. C. Liu C. T. (2014). A comparison of food-grade folium mori extract and 1-deoxynojirimycin for glycemic control and renal function in streptozotocin-induced diabetic rats. J. Tradit. Complement. Med. 4, 162. 10.4103/2225-4110.131639 25161921 PMC4142454

[B24] Huang X. L. He Y. Ji L. L. Wang K. Y. Wang Y. L. Chen D. F.. (2017). Hepatoprotective potential of isoquercitrin against type 2 diabetes-induced hepatic injury in rats. Oncotarget 8, 101545. 10.18632/oncotarget.21074 29254185 PMC5731895

[B25] Hunyadi A. Liktorbusa E. Márki Á. Martins A. Jedlinszki N. Hsieh T. J.. (2013). Metabolic effects of mulberry leaves: exploring potential benefits in type 2 diabetes and hyperuricemia. Evid. Based Complement. Alternat. Med. 2013, 948627. 10.1155/2013/948627 24381639 PMC3870074

[B26] Hunyadi A. Martins A. Hsieh T. J. Seres A. Zupkó I. (2012). Chlorogenic acid and rutin play a major role in the *in vivo* anti-diabetic activity of morus alba leaf extract on type II diabetic rats. PLoS ONE 7:e50619. 10.1371/journal.pone.0050619 23185641 PMC3503931

[B27] Ji T. Li J. Su S. L. Zhu Z. H. Guo S. Qian D. W.. (2016). Identification and determination of the polyhydroxylated alkaloids compounds with Î±-glucosidase inhibitor activity in mulberry leaves of different origins. Molecules 21, 206. 10.3390/molecules21020206 26867190 PMC6274138

[B28] Jouad H. Haloui M. Rhiouani H. Hilaly J. E. Eddouks M. (2001). Ethnobotanical survey of medicinal plants used for the treatment of diabetes, cardiac and renal diseases in the North centre region of Morocco (Fez–Boulemane). J. Ethnopharmacol. 77, 175. 10.1016/S0378-874100289-6 11535361

[B29] Katsube T. Imawaka N. Kawano Y. Yamazaki Y. Shiwaku K. Yamane Y. (2006). Antioxidant flavonol glycosides in mulberry (*Morus alba* L.) leaves isolated based on LDL antioxidant activity. Food Chem. 97, 25–31. 10.1016/j.foodchem.2005.03.019

[B30] Król E. Jeszka-Skowron M. Krejpcio Z. Flaczyk E. Wójciak R. W. (2016). The effects of supplementary mulberry leaf (*Morus alba*) extracts on the trace element status (Fe, Zn and Cu) in relation to diabetes management and antioxidant indices in diabetic rats. Biol. Trace Elem. Res. 174, 1–8. 10.1007/s12011-016-0696-1 27071614 PMC5055558

[B31] Kwak S. H. Park K. S. (2016). Recent progress in genetic and epigenetic research on type 2 diabetes. Exp. Mol. Med. 48, e220. 10.1038/emm.2016.7 26964836 PMC4892885

[B32] Li Y. Ji D. Zhong S. Lv Z. Lin T. Chen S.. (2011). Hybrid of 1-deoxynojirimycin and polysaccharide from mulberry leaves treat diabetes mellitus by activating PDX-1/insulin-1 signaling pathway and regulating the expression of glucokinase, phosphoenolpyruvate carboxykinase and glucose-6-phosphatase in alloxan-induced diabetic mice. J. Ethnopharmacol. 134, 961–970. 10.1016/j.jep.2011.02.009 21333726

[B33] Liao B. Y. Hu H. M. Thakur K. Chen G. H. Li L. Wei Z. J. (2018). Hypoglycemic activity and the composition analysis of the polysaccharide extracted from the fruit of mori multicaulis. Curr. Top. Nutraceutical Res. 16, 1–8.

[B34] Liu Q. Li X. Li C. Zheng Y. Peng G. (2015). 1-Deoxynojirimycin alleviates insulin resistance via activation of insulin signaling PI3K/AKT pathway in skeletal muscle of db/db mice. Molecules 20, 21700. 10.3390/molecules201219794 26690098 PMC6331926

[B35] Liu Y. Li X. Xie C. Luo X. Bao Y. Wu B.. (2016). Prevention effects and possible molecular mechanism of mulberry leaf extract and its formulation on rats with insulin-insensitivity. PLoS ONE 11:e0152728. 10.1371/journal.pone.0152728 27054886 PMC4824359

[B36] Michels A. W. Eisenbarth G. S. (2011). Immune intervention in type 1 diabetes. Semin. Immunol. 23, 214. 10.1016/j.smim.2011.07.003 21852151 PMC3177994

[B37] Mlinar B. Marc J. Janez A. Pfeifer M. (2007). Molecular mechanisms of insulin resistance and associated diseases. Clin. Chim. Acta 375, 20–35. 10.1016/j.cca.2006.07.005 16956601

[B38] Mu Q. Wang L. Yu F. Gao H. Lei T. Li P.. (2015). Imp2 regulates GBM progression by activating IGF2/PI3K/Akt pathway. Cancer Biol. Ther. 16, 623. 10.1080/15384047.2015.1019185 25719943 PMC4622833

[B39] Nie X. Q. Zhang D. D. Zhang H. Pharmacy S. O. University Z. M. (2017). Inflammation, insulin resistance and traditional chinese medicine treatment of diabetes mellitus. Chin. Pharmaceutical J. 1:2017.

[B40] Odermarsky M. Pesonen E. Sorsa T. Lernmark Å. Pussinen P. J. Liuba P. (2017). HLA, infections and inflammation in early stages of atherosclerosis in children with type 1 diabetes. Acta Diabetol. 55, 1–7. 10.1007/s00592-017-1063-1 29064046 PMC5794827

[B41] Okamoto R. M. Sagawa C. Taki M. Egashira F. Kasuga K. Kawamura W. (2007). Comparison with glimepiride and glibenclamide on gycogen accumulation in liver. Diabetes 56:pA677.

[B42] Ong S. Hall A. Dongworth R. Kalkhoran S. Pyakurel A. Scorrano L.. (2015). Akt protects the heart against ischaemia-reperfusion injury by modulating mitochondrial morphology. Thromb. Haemost. 113, 513–521. 10.1160/TH14-07-0592 25253080

[B43] Ong S. Katwadi K. Kwek X. Ismail N. Chinda K. Ong S.. (2018). Non-coding RNAs as therapeutic targets for preventing myocardial ischemia-reperfusion injury. Expert Opin. Ther. Targets 22, 247–261. 10.1080/14728222.2018.1439015 29417868

[B44] Ou T. Hsu M. Chan K. Huang C. Ho H. Wang C. (2011). Mulberry extract inhibits oleic acid-induced lipid accumulation via reduction of lipogenesis and promotion of hepatic lipid clearance. J. Sci. Food Agric. 91, 2740–2748. 10.1002/jsfa.4489 22002411

[B45] Parkkola A. Laine A. P. Karhunen M. Härkönen T. Ryhänen S. J. Ilonen J.. (2017). HLA and non-HLA genes and familial predisposition to autoimmune diseases in families with a child affected by type 1 diabetes. PLoS ONE 12:e0188402. 10.1371/journal.pone.0188402 29182645 PMC5705143

[B46] Pelantová H. Bugánová M. Holubová M. Šedivá B. Zemenová J. Sýkora D.. (2016). Urinary metabolomic profiling in mice with diet-induced obesity and type 2 diabetes mellitus after treatment with metformin, vildagliptin and their combination. Mol. Cell. Endocrinol. 431, 88–100. 10.1016/j.mce.2016.05.003 27164444

[B47] Peng C. H. Liu L. K. Chuang C. M. Chyau C. C. Huang C. N. Wang C. J. (2011). Mulberry water extracts possess an anti-obesity effect and ability to inhibit hepatic lipogenesis and promote lipolysis. J. Agric. Food Chem. 59, 2663–2671. 10.1021/jf1043508 21361295

[B48] Proença C. Freitas M. Ribeiro D. Jlc S. Carvalho F. Ams S.. (2017). Inhibition of protein tyrosine phosphatase 1B by flavonoids: a structure–activity relationship study. Food Chem. Toxicol. 111, 474–481. 10.1016/j.fct.2017.11.039 29175190

[B49] Reed M. J. Meszaros K. Entes L. J. Claypool M. D. Pinkett J. G. Gadbois T. M.. (2000). A new rat model of type 2 diabetes: the fat-fed, streptozotocin-treated rat. Metab. Clin. Exp. 49, 1390–1394. 10.1053/meta.2000.17721 11092499

[B50] Ren Q. Xiao D. Han X. Edwards S. L. Wang H. Tang Y.. (2016). Genetic and clinical predictive factors of sulfonylurea failure in patients with type 2 diabetes. Diabetes Technol. Ther. 18, 586. 10.1089/dia.2015.0427 27403931

[B51] Rubin A. Salzberg A. C. Imamura Y. Grivitishvilli A. Tombrantink J. (2016). Identification of novel targets of diabetic nephropathy and PEDF peptide treatment using RNA-seq. BMC Genomics 17:936. 10.1186/s12864-016-3199-8 27855634 PMC5114726

[B52] Sarikaphuti A. Nararatwanchai T. Hashiguchi T. Ito T. Thaworanunta S. Kikuchi K.. (2013). Preventive effects of *Morus alba* L. anthocyanins on diabetes in Zucker diabetic fatty rats. Exp. Ther. Med. 6, 689–695. 10.3892/etm.2013.1203 24137248 PMC3786992

[B53] Sheng Y. Zheng S. Ma T. Zhang C. Ou X. He X.. (2017). Mulberry leaf alleviates streptozotocin-induced diabetic rats by attenuating NEFA signaling and modulating intestinal microflora. Sci. Rep. 7, 12041. 10.1038/s41598-017-12245-2 28935866 PMC5608946

[B54] Shibata Y. Kume N. Arai H. Hayashida K. Inuihayashida A. Minami M.. (2007). Mulberry leaf aqueous fractions inhibit TNF-alpha-induced nuclear factor kappaB (NF-kappaB) activation and lectin-like oxidized LDL receptor-1 (LOX-1) expression in vascular endothelial cells. Atherosclerosis 193, 20. 10.1016/j.atherosclerosis.2006.08.011 17055514

[B55] Sia C. Weinem M. (2005). Genetic susceptibility to type 1 diabetes in the intracellular pathway of antigen processing–a subject review and cross-study comparison. Rev. Diabet. Stud. 2, 40–52. 10.1900/RDS.2005.2.40 17491658 PMC1762495

[B56] Song W. Wang H. J. Bucheli P. Zhang P. F. Wei D. Z. Lu Y. H. (2009). Phytochemical profiles of different mulberry (Morus sp.) species from China. J. Agric. Food Chem. 57, 9133–9140. 10.1021/jf9022228 19761189

[B57] Su R. Wang C. Feng H. Lin L. Liu X. Wei Y.. (2016). Alteration in Expression and methylation of IGF2/H19 in placenta and umbilical cord blood are associated with macrosomia exposed to intrauterine hyperglycemia. PLoS ONE 11:e0148399. 10.1371/journal.pone.0148399 26840070 PMC4739655

[B58] Toshiyuki K. Kiyotaka N. Hiroyuki K. Yoshihiro K. Yuko G. Kenji Y. (2007). Food-grade mulberry powder enriched with 1-deoxynojirimycin suppresses the elevation of postprandial blood glucose in humans. J. Agric. Food Chem. 55, 5869–5874. 10.1021/jf062680g 17555327

[B59] Wu T. Yin J. Zhang G. Long H. Zheng X. (2016). Mulberry and cherry anthocyanin consumption prevents oxidative stress and inflammation in diet-induced obese mice. Mol. Nutr. Food Res. 60, 687–694. 10.1002/mnfr.201500734 26627062

[B60] Yan F. Dai G. Zheng X. (2016). Mulberry anthocyanin extract ameliorates insulin resistance by regulating PI3K/AKT pathway in HepG2 cells and db/db mice. J. Nutr. Biochem. 36, 68. 10.1016/j.jnutbio.2016.07.004 27580020

[B61] Yang S. Deng H. Zhang Q. Jing X. Hui Z. Jin X. (2016). Amelioration of diabetic mouse nephropathy by catalpol correlates with down-regulation of Grb10 expression and activation of insulin-like growth factor 1/insulin-like growth factor 1 receptor signaling. PLoS ONE 11:e0151857 10.1371/journal.pone.0151857 26986757 PMC4795681

[B62] Yi H. Yi L. He R. Lv Q. Ren X. Zhang Z. (2012). Dynamic metabolic profiling of urine from type 2 diabetic KK-Ay mice treated with repaglinide by GC-MS. Anal. Lett. 45, 1862–1874. 10.1080/00032719.2012.677977

[B63] Yu H. X. Thai A. C. Chan S. H. (1999). HLA microsatellite associations with insulin-dependent diabetes mellitus in Singaporean Chinese. Hum. Immunol. 60, 894–900. 10.1016/S0198-885900071-3 10527399

