# Supplementary material for: Mulberry Leaf Regulates Differentially Expressed Genes in Diabetic Mice Liver Based on RNA-Seq Analysis
Source: Front Physiol. 2018 Aug 7;9:1051. doi: 10.3389/fphys.2018.01051 (PMC6090096; doi:10.3389/fphys.2018.01051)
Supplement: Supplementary file 1 [file Presentation_1.zip › supplementary materials/supplementary materials.docx]

**Table S1.** Effect of mulberry powder on the body weight of the diabetic model mice (x±s, g). Value= mean±SD (N=6)

| Time | Weight before modeling | Model 72 h weight | Weight 10 weeks after gavage |
| --- | --- | --- | --- |
| C | 28.86 ± 3.20 | 29.82 ± 2.20 | 33.02 ± 1.95 |
| DC | 28.25 ± 2.75 | 24.07 ± 2.77 | 25.83 ± 1.93 |
| DD | 28.79 ± 2.89 | 25.64 ± 1.48 | 29.19 ± 2.73 |

a P<0.05, DC compared with C

b P<0.05, DD compared with DC

**Table S2.** Glucose tolerance test (GTT, mmol/L) of all animals. Value= mean±SD (N=6)

| Time (h) | 0 | 0.5 | 1 | 1.5 | 2 |
| --- | --- | --- | --- | --- | --- |
| C | 6.3±0.3 | 12.8±0.3 | 8.1±1.0 | 7.2±0.5 | 6.4±1.1 |
| DC | 23.1±3.7^a^ | 24.8±1.4^a^ | 24.7±0.2^a^ | 24.6±4.1^a^ | 22.4±2.1^a^ |
| DD | 20.2±1.4^b^ | 23.9±4.5^b^ | 24.0±2.0^b^ | 22.5±1.6^b^ | 21.1±3.4^b^ |

a P<0.05, DC compared with C

b P<0.05, DD compared with C

**Table S3.** Fasting blood glucose levels (mmol/L) of the model mice (‾x±s, mmol / L). Value= mean±SD (N=6)

| Time | FBG | 0 d | 3 d | 2 w | 4 w | 10 w |
| --- | --- | --- | --- | --- | --- | --- |
| C | 6.7±1.1 | 7.10±0.61 | 6.80±0.74 | 6.6±0.37 | 6.9±0.48 | 6.73±0.25 |
| DC | 6.6±0.5 | 21.53±1.17^a^ | 22.77±3.20 ^a^ | 22.6±1.63 ^a^ | 20.6±5.06 ^a^ | 25.50±13.51^a^ |
| DD | 6.1±1.2 | 21.05±6.74^b^ | 20.32±2.02 ^b^ | 19.4±1.38 ^b^ | 13.9±2.43 ^ab^ | 10.73±2.37 ^ab^ |

a P<0.05, DC compared with C

b P<0.05, DD compared with DC

**Table S4.** Effect of mulberry leaf powder on serum insulin levels in the diabetic model mice (mU/L). Value= mean±SD (N=6)

|  | Before gavage | After 10 weeks gavage |
| --- | --- | --- |
| C | 10.94 ± 2.67 | 10.00 ± 1.69 |
| DC | 9.61 ± 0.44^a^ | 8.71 ± 1.56^a^ |
| DD | 10.12 ± 1.53^b^ | 10.91 ± 2.05^b^ |

a P<0.05, DC compared with C

b P<0.05, DD compared with DC

**Table S5.** qRT-PCR Identification of Differential Expressed Gene Primer Sequence (5'-3 ')

| Gene | ENSEMBL# | Primer sequence（5’-3’） |
| --- | --- | --- |
| *Scube1* | ENSMUSG00000016763 | F： CACTCTACGGGACAACCCAC |
|  |  | R：CACCCGTTCATCTCCACACA |
| *Alas2* | ENSMUSG00000025270 | F：GCAGCTATGTTGCTACGGTC |
|  |  | R：GATGGGGCAGCGTCCAATAC |
| *Cyp4a12b* | ENSMUSG00000078597 | F：ACAGATTTCTAGCTCCCTGGATTG |
|  |  | R：TGCCATGATTTCCGTGTAAGG |
| *Elfn1* | ENSMUSG00000048988 | F：CCGCTGGTGATCCTGACTAC |
|  |  | R：CGTGTACATGCGGTTGAACG |
| *Kif18b* | ENSMUSG00000051378 | F：TGTGATCAGCGTGGACCATC |
|  |  | R：GGAATGCTCAACGTGGGAGG |
| *Lrtm2* | ENSMUSG00000055003 | F：GGTTCTCCTACAGAGGGGGT |
|  |  | R：GTGGTCCTGGAGCATCTAGC |
| *Cd300e* | ENSMUSG00000048498 | F：AAGAAAAGAGGAGTGGCCCG |
|  |  | R：GCACCAGTAAGACCCAGCAT |
| *Amn* | ENSMUSG00000021278 | F：CAACTGGAACCAGAACCGGA |
|  |  | R：AGCATATCCGAGATGGCGTG |
| *Them7* | ENSMUSG00000055312 | F：TGTGTGTTACCGACTGGCTC |
|  |  | R：CGTGGTACATGCCAGAGTGT |
| *Pnpla5* | ENSMUSG00000018868 | F：CCTGGAGAGACGGGGACTTA |
|  |  | R：TGACAGTTGGGCCAGTCTTC |
| *Ccdc69* | ENSMUSG00000049588 | F：CGCAGACATCGAATGCACAG |
|  |  | R：ACCCGTCCACCTTGTTCATC |
| *Gulp1* | ENSMUSG00000056870 | F：AGATCTGCTGAGACCAAACGG |
|  |  | R：ACCTGCTGTCTAATGGGTCG |
| *Spns3* | ENSMUSG00000020798 | F：CTGTCTTGTAACTGGGCGGT |
|  |  | R：ACTATGGCGAAGGCACAACA |
| *Cidea* | ENSMUSG00000024526 | F：CCTACGACATCCGATGCACA |
|  |  | R：CATGAACCAGCCTTTGGTGC |
| *Ubd* | ENSMUSG00000035186 | F：TCCTCAAGCCCCATCGAAAA |
|  |  | R：TCTTCGAACTCGGAGGAGGT |
| *Scgb1c1* | ENSMUSG00000038801 | F：ACCCCGGAAGAACTCTACGA |
|  |  | R：CAGCTTGACCAGTTGCTCCT |
| *Ly6c1* | ENSMUSG00000079018 | F：GCAGTGCTACGAGTGCTATGG |
|  |  | R：ACTGACGGGTCTTTAGTTTCCTT |
| *Prrt3* | ENSMUSG00000045009 | F：AGATGAAGCCGAGGATTGGC |
|  |  | R：CCAGAGCTCTCTGTGTGGTG |
| *Trim5* | ENSMUSG00000060441 | F：CCATCGCCAGGGAACAAAGA |
|  |  | R：GTCACCTGAACCCAGTAGCG |
| *Ly6a* | ENSMUSG00000075602 | F： AGGAGGCAGCAGTTATTGTGG |
|  |  | R：CGTTGACCTTAGTACCCAGGA |
| *Ckap2* | ENSMUSG00000037725 | F：GACCCGGCATGTCACCATAA |
|  |  | R：ACTCTCGCCTTTGACACTGG |
| *Sult2a7* | ENSMUSG00000094156 | F：GTCAGGAATGACCTGGCTTGT |
|  |  | R：GGTCATGAGTCGTGGTCCTT |
| *Dnase1* | ENSMUSG00000005980 | F：AGGGCTAATGGGAACACTGC |
|  |  | R：GGTGTCAGGTTTGTCCCGAT |
| *Psrc1* | ENSMUSG00000068744 | F：AAATGAAGTAGCTCCCGCCC |
|  |  | R：CCGATCTTTCAGGGCACACT |
| *Ccnb2* | ENSMUSG00000032218 | F：GCTAGCTCCCAAGGATCGTC |
|  |  | R：CTGCAGAGCTGAGGGTTCTC |
| *Arhgef39* | ENSMUSG00000051517 | F：TCCTTTTCTCCGACGTGCTC |
|  |  | R：CCTGCTAAGGTGACACTGGG |
| *Grb10* | ENSMUSG00000020176 | F：GTCGTGGCTATTGTTGGATGG |
|  |  | R：CAGATGCCCTTGGTTTGAGA |
| *Tap1* | ENSMUSG00000037321 | F：GGTTCTCTTGATTCTCTCTTGCC |
|  |  | R：GTTGCGGGTGAAGCTAGGA |
| *Cyp51a1* | ENSMUSG00000001467 | F：TCAACTCAACGAGAAGGTGGCT |
|  |  | R：TCTATCCCTGCGCCTGAAA |
| *Igf2* | ENSMUSG00000048583 | F：CCGAGAGGGACGTGTCTAC |
|  |  | R：GTCTCCAGGTGTCATATTGGAAG |
| *Cck* | ENSMUSG00000032532 | F：TAGTCCCTGCAGAAGCTACG |
|  |  | R：CTTAAGAACGGACATGCGGC |

**Table S6.** Identification of Differential Expressed by qRT-PCR and RNA-Seq analysis. The left represents relative gene expression level measured by qRT-PCR, while on the right represents FPKM (fragments per kilobase of exon per million fragments mapped) of the gene measured by RNA-Seq for the same stages using the same RNA samples. All qRT-PCR data are shown as mean ± standard error; n=3.

| GENE | qRT-PCR (2^–△△CT^) | | | RNA-Seq (fpkm) | | |
| --- | --- | --- | --- | --- | --- | --- |
|  | C | DC | DD | C | DC | DD |
| *Scube1* | 1 | 2.489690946 | 0.644863924 | 33.2301 | 36.3698 | 32.009 |
| *Kif18b* | 1 | 2.253707053 | 0.573262044 | 0 | 0.149687 | 0.0121853 |
| *Cd300e* | 1 | 2.580767186 | 0.141107509 | 0.130278 | 2.88607 | 1.38129 |
| *Amn* | 1 | 2.264773332 | 0.502815141 | 0.0387972 | 0.775186 | 1.1299 |
| *Them7* | 1 | 24.45628447 | 11.5968949 | 1.02641 | 16.9406 | 19.5442 |
| *Pnpla5* | 1 | 4.656566774 | 3.413062106 | 0.657612 | 2.87298 | 0.630721 |
| *Cidea* | 1 | 0.318345807 | 1.582640228 | 0 | 0.503897 | 0 |
| *Trim5* | 1 | 0.861367025 | 1.215542217 | 0 | 0.343544 | 0 |
| *Sult2a7* | 1 | 12.14788569 | 39.21046013 | 0.644862 | 25.2815 | 6.29083 |
| *Dnase* | 1 | 1.762878852 | 4.020198303 | 0.0367142 | 0.775156 | 0.133562 |
| *Psrc1* | 1 | 0.683020128 | 1.120337442 | 0.0475619 | 0.715881 | 0.0875111 |
| *Ccnb2* | 1 | 0.494771167 | 4.940761218 | 0.0301659 | 1.61197 | 0.135987 |
| *Elfn1* | 1 | 0.433178437 | 0.436988333 | 0 | 0.104335 | 0.229244 |
| *Lrtm2* | 1 | 0.075657837 | 0.084609688 | 0 | 0.177602 | 0.319123 |
| *Gulp1* | 1 | 0.289439421 | 0.090166375 | 0.155114 | 0 | 0.0543955 |
| *Ccdc69* | 1 | 0.192335903 | 0.019709933 | 0.346901 | 0 | 0.0816499 |
| *Spns3* | 1 | 2.895207333 | 2.932707796 | 1.52399 | 1.52729 | 1.9583 |
| *Alas2* | 1 | 19.70969209 | 1.21993138 | 16.099 | 112.419 | 4.17322 |
| *Scgb1c1* | 1 | 2.272216022 | 0.953078312 | 0 | 5.42341 | 0.169539 |
| *Prrt3* | 1 | 0.172487852 | 0.658474739 | 0 | 0.0943622 | 0 |
| *Ckap2* | 1 | 0.238870401 | 0.339833504 | 0.0174504 | 1.34529 | 0.155495 |
| *Arhgef39* | 1 | 0.50019643 | 0.628855302 | 0.0307708 | 0.669846 | 0.0584395 |
| *Cck* | 1 | 2.374735225 | 0.04737052 | 2.57591 | 0 | 9.56117 |
| *Cyp4a12b* | 1 | 2.073018991 | 0.003118569 | 7.1065 | 34.1441 | 0.0191156 |
| *Ly6c1* | 1 | 7.413218322 | 0.188556223 | 1.38674 | 7.97779 | 0.146249 |
| *Ly6a* | 1 | 75.48405388 | 0.379884653 | 17.2049 | 500.751 | 4.86134 |
| *Grb10* | 1 | 6.05852528 | 0.214423262 | 1.59099 | 0.624646 | 0.855664 |
| *Tap1* | 1 | 6.619244982 | 0.642543782 | 4.9481 | 10.3776 | 1.29916 |
| *Cyp51A1* | 1 | 0.398844973 | 0.809143038 | 21.1893 | 8.36207 | 103.97 |
| *Igf2* | 1 | 93.51332185 | 6.424125156 | 0.0657565 | 0.985 | 0.107163 |
| *UBD* | 1 | 8.065701418 | 4.973862642 | 0 | 1.14817 | 0.0482086 |

**List of abbreviations**

| **Abbreviations** | **Full Name** |
| --- | --- |
| DGEs | differentially expressed genes |
| GO | Gene Ontology |
| KEGG | Kyoto Encyclopedia of Genes and Genomes |
| qRT-PCR | Quantitative real time polymerase chain reaction |
| IRS | Insulin receptor substrate |
| INS | Insulin |
| Scube1 | Signal peptide, CUB and EGF-like domain-containing protein 1 |
| Alas2 | 5-aminolevulinate synthase, erythroid-specific, mitochondrial |
| Cyp4a12b | Cytochrome P450, family 4, subfamily a, polypeptide 12B |
| Elfn1 | Extracellular leucine-rich repeat and fibronectin type III domain-containing 1 |
| Kif18b | Kinesin-like protein KIF18B |
| Lrtm2 | Leucine-rich repeat and transmembrane domain-containing protein 2 |
| Cd300e | CMRF35-like molecule 2 |
| Amn | Amnionless |
| Them7 | Thioesterase superfamily member 7 |
| Pnpla5 | Patatin-like phospholipase domain-containing protein 5 |
| Ccdc69 | Coiled-coil domain-containing protein 69 |
| Gulp1 | PTB domain-containing engulfment adapter protein 1 |
| Spns3 | Spinster homolog 3 |
| Cidea | Cell death activator CIDE-A |
| Ubd | Ubiquitin D |
| Scgb1c1 | Secretoglobin family 1C member 1 |
| Ly6c1 | Lymphocyte antigen 6C1 |
| Prrt3 | Proline-rich transmembrane protein 3 |
| Trim5 | Tripartite motif-containing protein 5 |
| Ly6a | Lymphocyte antigen 6A-2/6E-1 |
| Ckap2 | Cytoskeleton-associated protein 2 |
| Sult2a7 | Sulfotransferase |
| Dnase1 | Deoxyribonuclease-1 |
| Psrc1 | Proline/serine-rich coiled-coil protein 1 |
| Ccnb2 | G2/mitotic-specific cyclin-B2 |
| Arhgef39 | Rho guanine nucleotide exchange factor 39 |
| Grb10 | Growth factor receptor-bound protein 10 |
| Tap1 | Antigen peptide transporter 1 |
| Cyp51a1 | Lanosterol 14-alpha demethylase |
| Igf2 | Insulin-like growth factor II |
| Cck | Cholecystokinin |
| Sult2a7 | Sulfotransferase family 2A, dehydroepiandrosterone (DHEA)-preferring, member 7 |
| PPAR | peroxisome proliferators-activated receptors |
| AMPK | Adenosine 5‘-monophosphate (AMP)-activated protein kinase |
| TNF-α | Tumor necrosis factor-α |
